# Transgenic and genome-edited fruits: background, constraints, benefits, and commercial opportunities

**DOI:** 10.1038/s41438-021-00601-3

**Published:** 2021-07-17

**Authors:** Maria Lobato-Gómez, Seanna Hewitt, Teresa Capell, Paul Christou, Amit Dhingra, Patricia Sarai Girón-Calva

**Affiliations:** 1 Department of Crop and Forest Sciences, University of Lleida-Agrotecnio CERCA Center, Lleida, 25198 Spain; 2 Department of Horticulture, Washington State University, PO Box, 646414 Pullman, WA USA; 3 ICREA, Catalan Institute for Research and Advanced Studies, 08010 Barcelona, Spain

**Keywords:** Metabolic engineering, Molecular engineering in plants

## Abstract

Breeding has been used successfully for many years in the fruit industry, giving rise to most of today’s commercial fruit cultivars. More recently, new molecular breeding techniques have addressed some of the constraints of conventional breeding. However, the development and commercial introduction of such novel fruits has been slow and limited with only five genetically engineered fruits currently produced as commercial varieties—virus-resistant papaya and squash were commercialized 25 years ago, whereas insect-resistant eggplant, non-browning apple, and pink-fleshed pineapple have been approved for commercialization within the last 6 years and production continues to increase every year. Advances in molecular genetics, particularly the new wave of genome editing technologies, provide opportunities to develop new fruit cultivars more rapidly. Our review, emphasizes the socioeconomic impact of current commercial fruit cultivars developed by genetic engineering and the potential impact of genome editing on the development of improved cultivars at an accelerated rate.

## Introduction

The conventional breeding of fruit crops can take more than two decades due to the long juvenile period of woody species^1^. Genetic engineering allows improved varieties to be developed more quickly, and the vegetative propagation of fruit trees allows the engineered cultivars to achieve coverage of larger areas than crops that depend on sexual reproduction^2^. All genetically engineered fruit crops have been produced either by *Agrobacterium*-mediated transformation or direct DNA transfer. In each case, the efficiency of transformation is highly dependent on the species and even cultivar, requiring the development of bespoke optimized methods consisting of efficient gene delivery, selection, and regeneration from transformed explants^2^. Most fruit tree species are highly heterozygous, and to maintain the characteristics of the original variety the transgenic events should be derived from mature tissue (such as leaves) rather than embryogenic explants^3^.

The first genetically engineered fruit product (Flavr Savr™ tomato) was deregulated in 1992 and introduced into the market in 1994^4^. A gene that triggers pectin solubilization was downregulated in the transgenic fruits, resulting in delayed fruit softening and an extended shelf-life^5^. Several additional fruit crops with traits improved by genetic engineering have received regulatory approval for commercialization in different parts of the world, and are intended for cultivation either as human food or animal feed. These are tomato (*Solanum lycopersicum*)^6–9^, papaya (*Carica papaya* L.)^10,11^, pepper (*Capsicum annuum*)^12^, plum (*Prunus domestica*)^13^, eggplant (*Solanum melongena* L.)^14^, apple (*Malus domestica* Borkh.)^15^, melon (*Cucumis melo* L.)^16^, and pineapple (*Ananas comosus* L. Merr.)^17^. Most of the transgenic fruits were developed to improve agronomic productivity by conferring pest or disease resistance, or delayed ripening. However, more recent products have addressed quality traits by eliminating fruit browning or adding new visual traits such as flesh color. Some engineered fruit crops have been withdrawn from the market because they were not commercially viable (Flavr Savr™ tomato^4,18^) or were never commercialized (Melon A and B^16,19^).

Advances in genetic engineering, particularly the development of genome editing technologies have provided new tools for the generation of improved fruit varieties. Many proof-of-concept examples involving fruit crops have been reported and the further development and marketing of such varieties could have a major socioeconomic impact. Here we discuss the history and current status of genetically engineered fruit crops and the promise offered by genome editing. In recent years, several countries have amended their current regulations or have developed new guidelines to regulate genome-edited plants and its products^20^. This may make it possible that genome-edited fruits, similarly to all other genome-edited crops, reach the market faster in countries with a genome editing friendly policy^20,21^. Here, we first discuss fruit varieties that have already been approved for commercialization, focusing on those that are on the market. We then consider fruit varieties developed more recently using genetic engineering or genome editing, and their potential socioeconomic impact.

## Genetically engineered fruits approved for commercialization

### Trait description and drivers

**Fig. 1:**
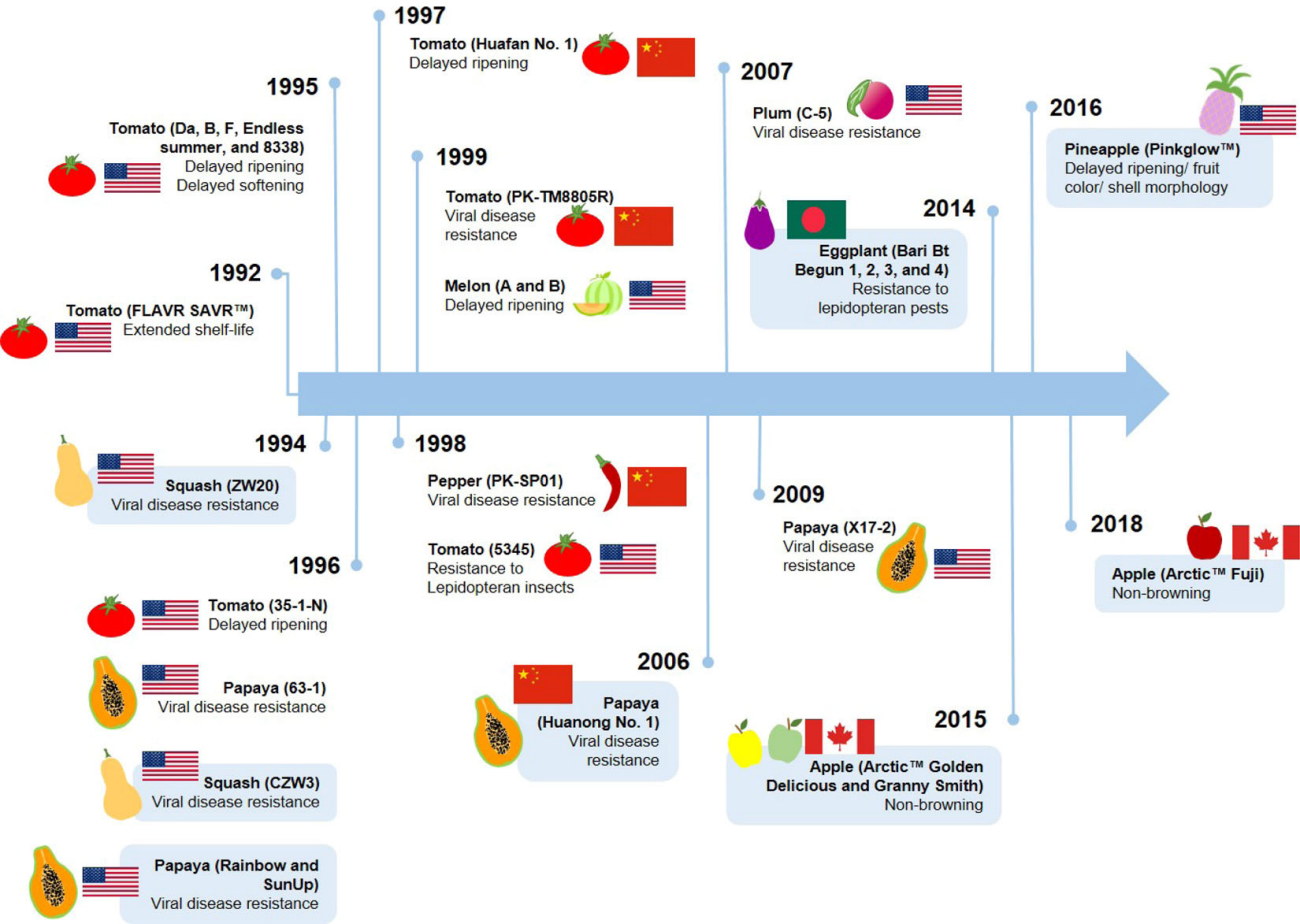
Timeline of development of fruit crops with engineered traits. Year indicates the year of first approval. Currently on the market indicated as light blue boxes

Genetically engineered fruits have been developed with unique agronomic characteristics that are often difficult to achieve by conventional breeding, and are designed to meet the specific needs of growers and/or customers. Fruits that have been developed by genetic engineering are shown in Fig. 1. Some varieties were approved but not ultimately commercialized, or were launched but subsequently removed from the market, and these are not considered in detail.

#### Papaya resistant to papaya ringspot virus

In 1992, papaya ringspot virus (PRSV) was detected in Puna, the major papaya-producing district in Hawaii. PRSV resistance was not found in papaya germplasm or in wild *Carica* species suitable as candidates for interspecific hybridization. Furthermore, insecticides failed to control the aphid vectors responsible for virus transmission^22^, and many orchards were therefore abandoned due to PRSV infestation^10^. The widely cultivated ‘Sunset’ papaya was transformed with a gene derived from a Hawaiian strain to produce the transgenic papaya ‘SunUp’, which is completely resistant to PRSV in Hawaii^10^. ‘SunUp’ papaya was crossed with ‘Kapoho’, a non-engineered cultivar, to obtain the yellow-flesh ‘Rainbow’ papaya, which is also resistant to PRSV^23^.

In China, PRSV has threatened the papaya industry for 50 years^24^. Similarly to the ‘SunUp’ variety, transgenic Huanong No. 1 papaya is resistant to the four predominant PRSV strains found in South China (Hainan, Guangdong, Guangxi, and Yunnan provinces), namely Ys, Vb, Sm and Lc^24^. Additionally, Huanong No. 1 produces bigger fruits with thicker flesh than the original cultivar^24^. In 2012, some Huanong No. 1 papayas grown in Hainan exhibited PRSV-like symptoms, suggesting that resistance is beginning to break. Phylogenetic analysis revealed the presence of a new virus lineage in Hainan and Guangdong papaya plantations, which may pose a threat to Huanong No. 1 papaya cultivation^25^.

#### Tomato and sweet pepper resistant to cucumber mosaic virus

In 1990, tomato crops in Fujian province (China) were affected by a virulent strain of cucumber mosaic virus (CMV) causing severe necrosis^26^. CMV is a major threat to tomato and sweet pepper and thus the tomato line PK-TM8805R and the sweet pepper line PK-SP01 were developed^24^. Both fruits express a CMV protein gene, conferring resistance to CMV, but data concerning the performance of these cultivars have not been published^26^.

#### Squash resistant to potyviruses

Like CMV, zucchini yellow mosaic virus (ZYMV) and watermelon mosaic virus 2 (WMV 2) are potyviruses transmitted by aphids. Together, these viruses can reduce the yields of squash by up to 80%^27^. Resistance to these viruses is not found in squash germplasm, and cannot be introduced by interspecific hybridization due to hybrid incompatibility and the concomitant transfer of undesirable traits^28^. In 1995, several transgenic inbred squash lines were developed by transformation with single or multiple viral protein genes from ZYMV, WMV2, and CMV. Transgenic lines ZW-20 and CZW-3 showed complete resistance to ZYMV and WMV2, line CZW-3 showed additional resistance to CMV^28^.

#### Eggplant resistant to eggplant fruit and shoot borer

In Bangladesh, eggplant is the second most important fruit crop and a major source of income for small, resource-poor farmers^29^. Eggplant fruits are unmarketable when infested with eggplant fruit and shoot borer (EFSB) larvae (*Leucinodes orbonalis*) but effective prevention requires the application of more than 100 sprays of insecticide each season. In addition to the detrimental impact on the environment, this accounts for more than a quarter of production costs, and there are still losses due to the prevalence of EFSB^30^. Resistant cultivars have not been developed by conventional breeding^31^, but a transgenic variety producing *Bacillus thuringiensis* (Bt) toxins is resistant to EFSB has been commercialized^30^. Infestations of the Bt variety occur at a frequency of 0.04–0.88% compared to 48–57% for the equivalent non-transgenic cultivar. In 2019, the average yield of Bt eggplant in Bangladesh was 19.8 t/ha, compared to 16.6 t/ha for the non-transgenic cultivar^29^.

#### Non-browning apple

Fruit quality is affected by the activity of polyphenoloxidases (PPOs), which oxidize phenolic compounds and cause gradual browning in fleshy fruits such as apple. PPOs are activated by exposure to oxygen, resulting in browning when fruits are damaged, peeled, or cut. Enzymatic browning can be prevented by storage in an air-free environment, the inactivation of PPOs by irradiation, or through the use of chemical inhibitors and natural antioxidants^32^. The Arctic^®^ apple concept was developed by silencing of PPOs^33,34^. Currently, there are three commercial varieties of Arctic^®^ apple: Arctic^®^ Golden Delicious, Arctic^®^ Granny Smith, and Arctic^®^ Fuji. Commercial harvest of Arctic^®^ Golden Delicious and Arctic^®^ Granny Smith started in 2016, and Arctic^®^ Fuji will be on the market in 2021^35^.

#### Pink-fleshed pineapple

Fruits with different skin and flesh colors have been developed by conventional breeding^36^ and in proof-of-concept engineering experiments^37^. In 2005, the Pinkglow™ transgenic pineapple was developed, in which the pink flesh accumulates lycopene due to the modification of the carotenoid pathway^17^. The skin of the Pinkglow™ pineapple also has a combination of green, yellow, orange, and red colors, whereas conventional pineapple is green and yellow. In addition to the modulation of carotenoid accumulation, an endogenous ethylene biosynthesis gene was suppressed to control flowering, but this trait has yet to be evaluated^17^.

### Development of commercial transgenic fruits (currently on the market)

In 1986, the coat protein of a Hawaiian PRSV isolate was cloned at Cornell University in collaboration with the Asgrow Seed Company. The USDA Section 406 grant program supported the development of transgenic PRSV-resistant papaya with the aim to control PRSV in Hawaii. In 1992, the first PRSV-resistant papayas were developed through a collaboration involving Cornell University, University of Hawaii and the Asgrow company^10^. The University of Hawaii established the protocol for papaya transformation by particle bombardment using zygotic embryos as the starting material^10,38^, whereas Huanong No. 1 papaya was generated using an *Agrobacterium*-mediated procedure established by an independent laboratory^11^. Transgenic papaya resistant to PRSV were developed using a pathogen-derived resistance approach, in which the resistance is mediated via RNA post-transcriptional gene silencing. The underpinning mechanism involves the expression of a partial or full pathogen gene sequence in the host to disrupt the pathogen’s replication^39^. ‘SunUp’ and ‘Rainbow’ papaya contain the coat protein gene from the mild PRSV HA 5-1 isolate^10^. The coat protein is required for virus survival outside the cell and for aphid transmission^40^. The required RNA specificity explains why PRSV-resistant transgenic papaya shows a narrow spectrum of resistance to particular PRSV isolates^41^. Huanong No.1 contains the replicase protein domain (NIb) from the PRSV Ys isolate, the most prevalent strain in China in 1994^24^. The N1b and N1a proteins are needed for virus replication^40^.

Seminis Vegetable Seeds and Monsanto Company developed transgenic virus-resistant squashes in 1995^27^. ZW-20 and CZW-2 virus-resistant squashes were generated using an *Agrobacterium*-mediated transformation protocol^28^ PTGS has been also used to produce ZW-20 and CZW-3 squash. Specifically, these lines contain the coat protein gene from FL isolates of ZYMV and WMV2, and line CZW-3 contains in addition the coat protein gene from CMV strain C^28^.

In 2000, the Maharashtra Hybrid Seeds Company (Mahyco) started to develop Bt eggplant with the collaboration of Monsanto, in India. In 2003, the Agricultural Biotechnology Support Project II (ABSPII) funded a partnership between Mahyco, Cornell University, the US Agency for International Development (USAID), and public-sector partners in India, Bangladesh, and the Philippines to develop and commercialize Bt eggplant. Under the ABSPII agreement, the EE-1 eggplant event, resistant to EFSB, was donated to the public Bangladesh Agricultural Research Institute (BARI) by Mahyco via a public–private partnership^30^. EFSB resistance was incorporated into nine local eggplant lines by BARI. The ASBPII project ended in 2014 and the distribution of Bt eggplant to farmers in Bangladesh was funded by the South Asia Eggplant Improvement Partnership (SAEIP), which comprises BARI, Cornell University, USAID, the University of the Pihilippines Los Banos, and Allience for Science^14,30^. Mahyco also set up its own eggplant transformation pipeline. Cotyledons from eggplant seedlings were used as explants for *Agrobacterium*-mediated transformation with the Bt *cry1Ac* gene, producing the EE-1 transgenic variety^42^.

Okanagan Specialty Fruits developed Arctic^®^ Apple events GD743 (Golden Delicious), GS784 (Granny Smith)^33^ and GS784 (Fuji)^35^ using their patented method to limit quinone biosynthesis^43^. Quinones are produced from diphenols in a reaction catalyzed by PPO, and their condensation with amino acids and proteins generates lignin-like compounds that cause browning. Cell damage is needed for plastidial PPO to act on vacuolar substrates, which is why browning only occurs in cut or otherwise damaged fruit^43^. RNA interference (RNAi) technology was used to target four apple PPO genes by expressing a chimeric sense RNA containing partial coding sequences of *PPO2*, *GPO3*, *APO5* and *pSR7*, leading to the generation of dsRNA and the suppression of homologous genes by post-transcriptional silencing^32^.

Del Monte started to develop the Pinkglow™ pineapple by modulating the carotenoid pathway^44^. ‘MD2’, also known as the Del Monte Gold pineapple, is a commercial variety developed by the company and was used as starting material. Ten years later, this transgenic pineapple was patented in the US^17^. Del Monte also patented the transformation method, which involved the cultivation of organogenic pineapple cells with *A. tumefaciens*. Conventional pineapple on the market has yellow flesh, reflecting the β-carotene content. The Pinkglow™ pineapple expresses the tangerine (*Citrus reticulata*) *PSY* gene, which is a rate-limiting enzyme in carotenoid biosynthesis during fruit development^17^. In addition, the endogenous lycopene β and ε cyclase genes (*βLYC* and *εLYC*) were suppressed by RNAi^17^. Ethylene promotes flowering in pineapple, and 1-aminocyclopropane-1 carboxylic acid (ACC) is the immediate ethylene precursor in plants^45^. A meristem-specific ACC synthase (ACS) was suppressed by RNAi in the Pinkglow™ pineapple to inhibit flowering^17^.

### Regulatory approval and commercialization of improved fruit crops

The USA has issued the most approvals for transgenic fruit cultivation either for human consumption or as animal feed. Like other genetically engineered crops, three government agencies are responsible for the oversight of transgenic fruit cultivation and import: the US Department of Agriculture (USDA) Animal and Plant Health Inspection Service (APHIS), the US Environmental Protection Agency (EPA), and the US Food and Drug Administration (FDA), which is part of the Department of Health and Human Services. Depending on its characteristics, a genetically engineered fruit may fall under the jurisdiction of one or more of these agencies^46^. APHIS regulates the environmental release of genetically engineered organisms that may pose a risk to plant health, the EPA oversees pesticides, including genetically engineered plants expressing plant incorporated protectants (PIP), and the FDA ensures the safety of all human food and animal feed (also from plant origin).

In 2020, APHIS published a revision of its 1987 biotechnology regulations^47^. The new framework, known as the SECURE rule (Sustainable, Ecological, Consistent, Uniform, Responsible, and Efficient) differs from the previous regulatory framework by focusing on an organism’s properties and not on the production method^47^.

Flavr Savr™ tomato developed by Monsanto Company was the first genetically engineered fruit to gain non-regulated status from APHIS and approval by the FDA^5,18^. Flavr Savr™ was also approved for import into Mexico in 1995 by the Federal Commission for the Protection against Sanitary Risk (COFEPRIS), a decentralized organ of the Mexican Secretariat of Health that oversees the safe release and import of genetically engineered plants^48^. COFREPIS also permitted the import of the engineered tomato varieties Da, B, F, and Endless summer. Similarly, in 1995 Health Canada and Agriculture and Agri-food Canada determined that the Flavr Savr™ tomato was safe for human consumption and did not pose risks as a plant pest^49^. In Canada, the Flavr Savr™ tomato was marketed under the brand name MacGregor, allowing consumers to make an informed choice^49^. Flavr Savr™ was removed from the market in 1997 because the fruits were less firm than expected and the costs of production were uncompetitive^18^.

APHIS deregulated additional engineered tomato lines in the 1990s, namely Da, B, F developed by Zeneca and Petoseed Company; 35-1-N developed by Agritope, Inc; and 5345 and 8338 “Endless summer” developed by the Monsanto Company^6–9,50^. These lines were also approved as food and feed. The Da, B, and F lines were intended for processing^4^. Between 1996 and 1999, more than 1.8 million cans derived from hybrids of the F line were sold in the UK^18^, but from 1998 onwards were no longer used as food ingredients^18^. In 2000 Health Canada also approved line 5345, which was resistant to insect pests, but it has not been released onto the market^51^.

In 1999, Agritope was granted FDA approval of the Melon A and B lines for use as food^16^. The company also requested the deregulation of these lines, but withdrew the APHIS petition the same year^19^, and neither line has been commercialized.

The Pinkglow™ pineapple received FDA approval in 2016 and was marketed for the first time in October 2020 by Fresh del Monte^52,53^. This cultivar is grown on a single farm in Costa Rica. The C5 plum (HoneySweet) developed by the US Department of Agriculture, which is resistant to plum pox virus (PPV), has also been deregulated by APHIS, approved by the FDA and registered by the EPA^54^. It was patented in the US in 2004, but no trees have been planted thus far and it is therefore not on the market. On request, the Agricultural Research Service (the research branch of the USDA) can freely provide a limited number of heat-treated bud wood samples to be used as a genetic resource for the breeding of PPV-resistant varieties^55^.

Genetically engineered squash has been on the US market for 25 years. CZW3 squash is also approved for import as food by Health Canada^56^. The cultivation of genetically engineered papaya in the US began in 1996, and the current predominant variety is ‘Rainbow’ because it has yellow fruit flesh favored by consumers^4^. Canada and Japan are the major importers of genetically engineered papaya produced in the US, although it is also approved for cultivation in Japan^57^. Two additional papaya lines resistant to PRSV were approved for cultivation by APHIS: 63-1 developed by Cornell University and the University of Hawaii^58^, and X17-2 developed by the University of Florida, respectively^59^. Neither lines have been commercialized^4^.

Arctic^®^ apples were developed by Okanagan Specialty Fruits Company in Canada, and the Golden Delicious, Granny Smith, and Fuji varieties have received approval for cultivation, human consumption and use as animal feed in both Canada and the US^15,60–62^. However, Arctic^®^ apples are only grown in the US, and it is unclear if Artic varieties are among the 206,259 tons of apples (including dried apples) imported to Canada, most of which are grown in the US^63,64^.

In China, the commercialization of all genetically engineered crops is regulated by the Ministry of Agriculture (MOA)^65^, with safety advice provided mainly by the Biosafety Management Division of the Center for Science and Technology Development (CSTD) and the National Biosafety Committee (NBC). The NBC can recommend safety certification based on product testing and field trials, but only the MOA can formally provide regulatory clearance^25^. After registration, genetically engineered crops can be cultivated and commercialized but approval for commercialization is only granted at the province/region level and not nationwide.

Huafan No. 1 tomato developed by Huazhong Agricultural University was the first genetically engineered fruit to be approved for cultivation, human consumption and use as animal feed in China, followed by Da Dong No. 9 (Institute of Microbiology, CAS) and PK-TM8805R (Beijing University) tomatos^26^. Huafan No. 1 and Da Dong No. 9 are no longer cultivated in China, and the status of PK-TM8805R is unclear^26^. Similarly, the genetically engineered sweet pepper PK-SP01 developed by Beijing University was approved for cultivation and for human consumption, but the extent of its cultivation is unclear^26^. PRSV-resistant papaya Huanong No. 1 was approved for cultivation in 2006 and is commercially available in China.

In Bangladesh, the National Committee on Biosafety (NCB) grants regulatory approvals for all genetically engineered crops, assisted by a Biosafety Core Committee (BCC)^66^. The eggplant varieties Bari Bt Begun 1, 2, 3, and 4 were approved for cultivation and food use in Bangladesh, and in 2020 they are the only genetically engineered fruit commercialized in this country^29,30^.

### Socioeconomic impact of commercialized fruits with improved traits

The socioeconomic impact of genetically engineered fruits is growing with the scale of cultivation, although less than 0.01% of the 185.43 million ha cultivated with genetically engineered crops in 2018 was represented by fruits^67^. Production and adoption rate details are provided in Table 1. PRSV-resistant papaya is the most widely cultivated genetically engineered fruit, followed by Bt eggplant, virus-resistant squash, Arctic^®^ apples, and Pinkglow™ pineapple.

**Table 1:** Production and adoption rates of genetically engineered fruits on the market. Adoption rate = ha of transgenic crop (dark orange)/total ha of crop (light orange)^a^

Fruit	Modified trait	Trade or event name	Production (ha)	Adoption rate
	Non-browning	Arctic™ Golden Delicious, Granny Smith, and Fuji Apples	500 (2019, US)	
	Resistance to papaya ringspot virus	Rainbow, SunUp	405 (2017, US)	
	Resistance to papaya ringspot virus	Huanong No. 1	7130 (2017, China)	
	Resistance to Eggplant fruit and shoot borer (*Leucinodes orbonalis*)	Bari Bt Begun 1, 2, 3 and 4	2400 (2017, Bangladesh)	
	Delayed ripening/senescence Fruit color Shell morphology	Pinkglow™	25 (2017, Costa Rica)	
	Resistance to cucumber mosaic cucumovirus, zucchini yellow mosaic potyvirus and watermelon mosaic potyvirus 2	CZW3 and ZW20	1000 (2017, US)	

^a^Data extracted from refs. ^4,57^

#### Virus-resistant fruits

China grew 9600 ha of PRSV-resistant papaya in 2018. Initial plantings took place in the southern Guangdong Province in 2006, but Hainan Island became the leading location for PRSV-resistant papaya production in 2017 (46%), followed by Guangdong (36%) and Guangxi (18%) provinces^57^. CMV-resistant sweet pepper and tomato have been cultivated in China since 1998 and 1999, respectively, in Beijing municipality and in Fujian and Yunnan provinces, but the scale of cultivation is unclear^26^. Data on the profitability of PRSV-resistant papaya have not been published by the Chinese authorities, so the socioeconomic impact is difficult to judge^68^.

In the US, PRSV-resistant papaya has been commercially grown in Hawaii since 1999 and it has prevented the collapse of the Hawaiian papaya industry due to the prevalence of PRSV in orchards of conventional varieties^23^. In 1992, when PRSV was first detected on Hawaii, the Puna district produced 95% of all Hawaiian papaya grown (~24,000 tons) but yields had fallen to ~12,000 tons in 1998. Two years after the introduction of the resistant variety, yields recovered to ~18,000 tons^23^. Although lower than 1992 levels, the lack of production was not caused by the virus but by the falling demand from Japan, resulting in the papaya cultivation area in Hawaii declining from more than 500 ha in 2015 to only 250 ha in 2018^4,67^. The shrinking Japanese market partly reflected the reluctance of retailers to handle genetically engineered products and partly the increased competition from Philippine papaya growers^4^. Nevertheless, the yield of genetically engineered papaya in 2018 was 17% higher than conventional papaya, with a net farm income gain of $2623/ha. Overall, the accumulated farm income benefit between 1999 and 2018 was $38.4 million^67^. Cultivation of PRSV-resistant papaya in Hawaii has also reduced the threat of PRSV in the Puna district, allowing papaya growers to cultivate non-transgenic varieties alongside the genetically engineered crop^23^.

Virus-resistant squash has been commercially grown in the US since 2004, mainly in Florida and Georgia. In 2018, virus-resistant squash was planted on 1000 ha, representing 6% of total squash production in the US^67^. The genetically engineered varieties achieve higher yields than conventional squash, resulting in a net gain to farmers of $10.1 million. Overall, the cumulative farm income benefit between 2004 and 2018 was $310.9 million^67^.

#### Insect-resistant fruit crops

Bt eggplant was first grown commercially in Bangladesh in 2014, and was cultivated on 2975 ha in 2018^67^. Eggplant is mostly grown by resource-poor farmers, who can obtain seed at no or minimal cost from three organizations: BARI, the Department of Agricultural Extension, and the Bangladesh Agricultural Corporation. Accordingly, the cost of this technology to the farmers is near zero^29^. The Bt eggplant was initially provided to 20 farmers, but by 2018, the variety had been adopted by 20,695 farmers^29^. Bt eggplant achieved 20% higher yields than conventional eggplant in 2018, and the enhanced quality resulted in a 10% increase in price. As a result, farm income has increased by $616–704/ha^29,67^.

As well as the direct income gains, Bt eggplant also helps to reduce pesticides. In 2016, farmers in 35 districts cultivating Bt eggplant spent 61% less on pesticides compared to farmers growing conventional varieties^69^. This difference solely represents the cost of pesticides to control EFSB because different chemicals are used to control other pests. However, the prevention of damage caused by EFSB also reduces infestations by secondary pests such as leaf-eating beetles, thrips, whitefly, mites, leaf wing bugs, and leaf roller, by 42–60%^70^.

#### Fruits with enhanced quality traits

Arctic^®^ apples were first planted in 2016 (70,000 trees planted over 80 ha). This had grown to 300,000 trees over 101 ha by 2018 and in 2019 the cultivated area exceeded 500 ha^71^. Although the profitability of growing this variety has not been made public, Okanagan Specialty Fruits states that Arctic^®^ apples are more suitable for mechanical harvesting and suffer less impact from finger bruising, bin rubs and other superficial damage, which results in higher packouts (an industry measure of fruit suitable for market) and therefore less waste, and similar benefits for retailers^72^. Furthermore, the Arctic^®^ Golden variety does not require warm packing, reducing the cost of production. Del Monte commercialized the Pinkglow™ pineapple in October 2020 so the socioeconomic impact of this variety will not be known until market data are available.

## Technological advances in gene functional analysis and genetic modification of fruits

Genetic engineering can be used to investigate the functions of genes and to exploit these functions for the improvement of traits such as biotic and abiotic stress tolerance, flowering time, ripening, fruit flavor, and nutrient content. In this section, we discuss genetic engineering and genome editing technologies that have been used for the enhancement of target traits in fruit crops, which may facilitate commercialization in the future (Table 2). Use of CRISPR and associated genome editing technologies for the development or enhancement of fruit crops may open the door to new commercial opportunities, potentially circumventing restrictions on GM crops in many parts of the world^20^. While marketability will vary by country, additional, transgene-free cultivars may be accessible to consumers in the near future^20,73,74^.

**Table 2:** Current status of improving fruits through molecular tools (until mid-2020).

Fruit	Trait	Modification strategy	G	F	Outcome
	Flowering time	OE, GE	✓		Early flowering^103^
	Fruit morphology	OE, GS	✓		Different color^38^
					Different shape^138^
	Quality improvement	GS		✓	Increased firmness^107^
	Plant morphology	OE	✓		Smaller trees^139^
					Dwarf tree^119^
	Disease resistance	OE, GE	✓	✓	Increased resistance to bacteria and fungi^76,77,99^
	Tolerance to abiotic stress	OE	✓		Increased tolerance to drought and cold stress^140^
					Increased tolerance to salinity^95^
	Plant morphology	GE	✓		Shorter trees^141^
	Disease resistance	GE, GS, OE	✓	✓	Increased resistance to bacteria and virus^79,89,142–144^
	Nutritional improvement	GE	✓		Increased carotenoid content^145^
	Flowering time	OE	✓		Early flowering^146^
	Fruit morphology	GS	✓		Smaller fruits^147^
	Disease resistance	GS	✓		Increased resistance to virus^148^
Citrus rootstock species	Plant morphology	OE, GS	✓		Shorter trees^149^
	Disease resistance	OE	✓		Increased resistance to bacteria^150^
	Tolerance to abiotic stress	OE	✓		Increased tolerance to drought stress^98^
	Flowering time	OE	✓		Early flowering^104^
	Disease resistance	GE, OE, DR	✓		Increased resistance to bacteria^78,82,83,87,88^
	Nutritional improvement	GS	✓		Increased carotenoid content^114^
	Disease resistance	OE	✓		Increased resistance to fungi^84,85^
	Disease resistance	OE	✓		Increased resistance to virus^151^
	Disease resistance	GE	✓^a^		Increased resistance to virus^93^
	Disease resistance	OE		✓	Increased resistance to virus^152^
	Fruit morphology	OE		✓	Reduce pathogen-induced mortality^120^
	Tolerance to abiotic stress	OE	✓		Different color^112^
					Increased tolerance to salinity^153^
					Increased tolerance to cold stress^100^
	Nutritional improvement	OE	✓		Increased carotenoid content^154^
	Quality improvement	GS	✓		Ripening^108^
	Tolerance to abiotic stress	OE	✓		Increased tolerance to salinity^96^
	Disease resistance	GS	✓		Increased resistance to virus^90^
	Quality improvement	GS		✓	Delayed fruit ripening^109^
	Quality improvement	OE, GS	✓		Decreased ethylene production^110^
	Disease resistance	OE	✓		Increased resistance to bacteria^86^
	Nutritional improvement	OE	✓		Increased tocopherol content^155^
	Disease resistance	OE	✓		Increased resistance to fungi^80^
	Tolerance to abiotic stress	OE	✓		Increased tolerance to salinity^97^
	Flowering time	OE	✓		Early flowering^105^
	Disease resistance	GS	✓		Increased resistance to virus^156^
	Flowering time	GE	✓		Early flowering^157^
	Nutritional improvement	GS	✓		Decreased starch and increased soluble sugar content^111^
					Increased anthocyanin content^113^
	Quality improvement	OE, GS	✓		Increased fruit firmness^158^
	Flowering time	GE	✓	✓	Early flowering^102^
	Quality improvement	GE	✓	✓	Increased shelf-life^159^
	Fruit morphology	OE, GE	✓		Parthenocarpic fruits^160^
	Nutritional improvement	GE	✓		Increased lycopene content^161^
	Disease resistance	OE, GE	✓		Increased resistance to bacteria^162^
	Insect resistance	OE	✓		Increased resistance to insect^94^
	Pest resistance	GE	✓		Increased herbicide resistance^122^
	Disease resistance	GS	✓		Increased resistance to virus^91^

*OE* overexpression, *GS* gene silencing, *GE* genome editing, *DR* down-regulation.

Stage of development: *G* greenhouse, *F* field trials.

A detailed list of modified genes and outcomes is provided in Table S1.

^a^Net-house.

### Pathogen and pest resistance

Pathogens and pests are severe constraints affecting the growth and development of fruit trees, the development and ripening of fruits, and the quality of fruit products. In 2017 up to 30% of the fruit and vegetables losses worldwide were pre-harvest, mainly caused by pests and pathogens^75^. In many cases, conventional breeding for resistance is not possible because strong resistance is not present in available germplasm and the introgression process would take too long^2^. One strategy to enhance disease resistance in fruit crops is the modification of receptors that directly interact with or perceive the presence of a specific pathogen. In apple, overexpression of the *HcrVf2* gene encoding such a receptor resulted in near-complete resistance to fungal scab (*Venturia inaequalis*)^76^. Recently, CRISPR/Cas9-mediated inactivation of the susceptibility-associated gene *DspA/E-interacting protein* (*DIPM4*), also encoding a receptor, significantly reduced bacterial fire blight (*Erwinia amylovora*) symptoms by 50% in apple^77^.

Another strategy for the mitigation of pathogen symptoms is the targeting of response pathways (innate immunity) in the host. For example, the *nonexpressor of pathogenesis-related 1* (*NPR1*) gene encodes a transcriptional regulator of pathogenesis-related (PR) protein genes as part of the salicylic acid-dependent systemically acquired resistance (SAR) pathway. Sweet orange trees (*Citrus sinensus*) overexpressing *NPR1* under the control of the phloem-specific *SUC2* promoter exhibited enhanced resistance to huánglóngbìng (citrus greening disease), and up to 46% of the engineered plants remained disease-free for 2 years^78^. These findings highlight the importance of promoter selection in overexpression studies and indicate that *NPR1* possesses a conserved role among tree fruit species in the response to pathogens.

Other PR-associated proteins have been targeted for modification in banana, chili pepper, and citrus in order to mitigate the effect of bacterial and fungal pathogens. In banana, the induction of a hypersensitive response (HR) by the overexpression of genes encoding an HR-assisting protein and a plant ferredoxin-like protein conferred resistance to banana *Xanthomonas* wilt, with 50–60% of the transgenic plants displaying no disease symptoms following inoculation^79^. Overexpression of the *pepper carboxylesterase* gene in chili pepper reduced infections by anthracnose fungus from 70% in wild-type plants to 20%^80^. Similarly, expressing the *J1-1* gene encoding an antifungal defensin reduced the frequency of anthracnose lesions by up to 90%^80,81^. CRISPR/Cas9 was used to inactivate the grapefruit *lateral organ boundary domain family protein 1* and orange *WRKY22* genes, which regulate immunity responses, improving resistance to canker caused by *Xanthomonas citri* subsp. citri (*Xcc*) in Duncan grapefruit (*Citrus* ✕ *paradisi*) and Wanjincheng orange (*Citrus sinensis* (L.) Osbeck)^82–85^. The CRISPR-induced mutation rate in grapefruit was 23–89%, and *Xcc* resistance was correlated with the mutation rate, as shown by the corresponding range of canker symptoms^85^. Similar findings were reported for orange plants with mutations in the *WRKY22* gene^83^.

In addition to the knockout of host genes to improve pathogen and pest resistance, pathogen-derived transgenes (or other heterologous genes) serve as additional routes for the improvement of fruit traits. In pear, the expression of a bovine lactoferrin gene, which encodes a bactericidal glycoprotein, reduced fire blight symptoms by 78% compared to controls^86^. In sweet orange, expression of the *E. amylovora* hairpin protein triggered HR in the host plants and reduced susceptibility to citrus canker by up to 79%^87^. The expression of a synthetic insect antimicrobial peptide (cecropin B) in blood orange improved long-term resistance to huánglóngbìng by 85–100%^88^.

An important strategy in the fight against viral diseases is the expression of non-translatable pathogen genes to elicit a PR response or to silence viral components essential for replication, packaging, or systemic spreading. RNAi-mediated silencing of viral components has been achieved in banana, resulting in the complete absence of bunchy top virus disease symptoms in transgenic plants 6 months after challenge^89^. Similarly, transgenic melon and watermelon (*Citrullus lanatus*) lines displayed up to 100% resistance when challenged with several cucurbit viruses^90,91^, and grafted transgenic plum lines remained resistant to PPV for more than 9 years^92^. In cucumber, the CRISPR/Cas9 system was used to mutate the *eukaryotic translation initiation factor 4E* gene, which is associated with CMV susceptibility, resulting in 100% virus-free fruits in the T3 generation^93^. Bt cry genes have been expressed in kiwifruit (*Actinidia chinensis*) and walnut (*Juglans regia*) to protect them against insect pests, resulting in 75–100% insect pest mortality^94^.

### Abiotic stress tolerance

Abiotic factors, such as drought, are also among the main factors causing pre-harvest losses of fruit and vegetables^75^. The engineering of abiotic stress tolerance in fruit trees allows them to be grown in environments where temperatures are sub-optimal, water is scarce, or high concentrations of salt and/or heavy metals in the soil are toxic and prevent the uptake of water and nutrients. Overexpression of the Na^+^/H^+^ cation antiporter gene *NHX1* in apple and kiwifruit prolonged survival in saline conditions by allowing the accumulation of higher concentrations of antioxidant flavonoids (60% more than normal) as well as sodium and potassium (2x more than normal) thus delaying the stress response^95,96^. In chili pepper, the expression of a tobacco osmotin gene increased yields by 31% accompanied by higher levels of proline, chlorophyll and reactive oxygen species (ROS) scavengers, as well as a higher relative water content^97^. Transgenic citrumelo (*Citrus paradise × Poncirus trifoliata*) plants overexpressing the enzyme Δ1-pyrroline-5-carboxylate synthase, required for proline synthesis, showed a 2.5-fold increase in drought tolerance, as determined by turgor pressure maintenance, stomatal conductance, photosynthetic rate, and transpiration rate^98^.

Fruit crops are often threatened by cold temperatures, which affect plant growth as well as the quality of maturing and ripening fruits. Cold tolerance is therefore an important target in commercial fruit development programs. In apple, overexpression of the transcription factor MYB4, which regulates cold-induced dormancy and stress pathways, allowed the transgenic plants to tolerate cold temperatures for long periods while maintaining normal water content, reflecting the accumulation of glucose, fructose, and sucrose to levels 30–38% higher than normal^99^. Overexpression of the Arabidopsis dehydration response element-binding 1b protein in grapevine reduced cold-induced wilting by 73%^100^. Similarly, the expression of a *Poncirus trifoliata* basic helix-loop-helix protein in pumello (*Citrus grandis*) enhanced cold tolerance, reduced electrolyte leakage by 13% and increased proline levels by up to 67% compared to wild-type plants^101^.

### Flowering time and dormancy release

Flowering time is a very important trait targeted for improvement in fruit crops because of its close association with the timing of fruit development. This trait is under strict genetic regulation and is dependent on environmental conditions, particularly temperature and day length, which limits the geographical regions in which crops can be cultivated^102^. Genetic engineering has been used to express floral activators or repressors, allowing the specification of floral transition and dormancy requirements in major fruit tree species. In transgenic apple, plum, and citrus trees, the overexpression of FT family floral activators needed to trigger bud breaking promoted early flowering (by up to 45 weeks in apple and 12 weeks in orange), and reduced dormancy requirements, eliminating them completely in plum^103–105^. Recently, CRISPR/Cas9 was used to inactivate the *self-pruning 5G* gene in tomato, which abolished sensitivity to day length and reduced the time to harvest by 2 weeks, translating to a greatly accelerated flowering stage and early fruit yield^102^. In kiwifruit, CRISPR/Cas9-mediated repression of the *CEN-like* genes also led to rapid and early terminal flowering^106^. These experiments provide insights into the genetic and environmental control of flowering time in different fruits and form the basis for additional engineering strategies to develop early or late-flowering cultivars adapted to specific growing regions.

### Fruit ripening and sensory attributes

The modulation of fruit ripening is one of the major strategies by which flavor, aroma, and nutrient profiles can be adjusted, and by which the shelf-life can be extended to improve marketability and reduce waste. In climacteric fruits such as apple, banana, and tomato, the key targets are genes associated with ethylene biosynthesis and degradation. In apple, the silencing of *ACS* and *ACC oxidase* (*ACO*) by expressing antisense RNA generated fruit that produced 60% less ethylene, increasing firmness by 20% and allowing cold storage for up to 3 years^107^. Although the synthesis of volatile esters was suppressed, sugar and organic acid accumulation were unaffected. Co-suppression and knockdown of ethylene-biosynthetic genes achieved similar results in pear, kiwifruit, and papaya^108–110^.

Sugar and organic acid content can be modified to enhance fruit flavor. In strawberry, the suppression of ADP-glucose pyrophosphorylase by expressing antisense RNA under the control of a fruit-specific promoter inhibited the conversion of sugar to starch and reduced the starch content of transgenic fruits by up to 47% while increasing the soluble sugar content by up to 37%^111^. Plant pigments such as anthocyanins and carotenoids are also major targets for metabolic engineering in fruits because they provide health benefits and allow the production of fruits with unique colors. The overexpression of MYB family transcription factors in apple, grapevine, and strawberry enhanced the production and storage of anthocyanins, with transgenic fruits accumulating up to 50% more than normal^36,112,113^. The accumulation of carotenoids has been achieved by the RNAi-mediated silencing of β-carotene hydroxylase in sweet orange, preventing conversion of β-carotene to xanthophylls and thus increasing the β-carotene content in the fruit pulp by 26-fold. *Caenorhabditis elegans* adults fed with diets supplemented with β-carotene-enriched orange pulp were 20% more resistant to hydrogen peroxide-induced oxidative stress than those fed with control diet^114^. These studies demonstrate how genetic engineering and genome editing can be used to produce fruits with enhanced flavor, texture, and nutrient levels.

### Trans-grafting

Grafting is widely used during the propagation of fruit trees to allow the selection of rootstock and scions with different favorable characteristics that may be difficult or laborious to combine in one cultivar (such as high fruit yields paired with resistance to root pests). The rootstock and scion still influence each other by exchanging soluble signals, but the two components maintain their genetic integrity^115^. Trans-grafting refers to grafting of a non-transgenic scion onto a transgenic rootstock. Some desirable characteristics of the rootstock, such as dwarfing or disease resistance, are conferred upon the scion by the vascular transport of RNA, hormones or signaling proteins, but the shoot, leaves, and fruits remain transgene-free^116,117^. Although the specific regulations vary by country, trans-grafting can be used to circumvent restrictions on the marketing of GM products in certain jurisdictions^118^. This technology has been used in apple, by grafting non-transgenic scions onto rootstock expressing the *Agrobacterium rhizogenes rolB* gene, which confers dwarfing characteristics on the scion^119^. In grapevine, non-transgenic scions were grafted onto rootstocks engineered to produce an antimicrobial peptide and a protein that inhibits cell wall degradation. These proteins were transported to the scion through the xylem, resulting in the enhanced mobilization of water and nutrients and a 30–95% reduction in pathogen-induced mortality^120^. Transgenic rootstocks can therefore improve the production of commercially important fruit trees but the fruits and seeds do not carry any exogenous DNA^79^.

## Moving beyond transgenesis—genome editing technologies

Genome editing is perhaps the most important recent development in crop breeding, and protocols based on the versatile CRISPR/Cas9 system have been optimized for several fruit species to increase the editing efficiency. In apple, CRISPR/Cas9 produced transgene-free edits^121^. In cucumber, wild strawberry, and watermelon, CRISPR/Cas9 constructs were integrated as part of the T-DNA but segregation was then achieved through back-crossing^122–125^. A major challenge to the commercial development of edited varieties is the successful transmission of targeted mutations through the germline^126^. This is particularly difficult in woody species, including fruit trees, because they are propagated vegetatively. Back-crossing could take decades (depending on the species) and could result in the unintentional outcrossing of the edited gene. It is also difficult to achieve homozygosity at the edited locus within the desired genetic background because most fruit trees are self-incompatible and thus require obligate outcrossing. Such characteristics hinder the introduction of genome edits that are stable and heritable^127–129^. Several new derivatives of the original CRISPR/Cas9 editing platform have been proposed, including CRISPR/Cas9 ribonucleoprotein (RNP) technology, CRISPR cytidine and adenosine base editors (CBEs/ABEs), CRISPR flippase, and new CRISPR-associated nucleases such as Cas12a/Cpf1, which may help to address these challenges and accelerate the development and commercialization of genome-edited crops^77,126,129–132^.

### CRISPR RNP technology

Transgene-free genome editing improves the commercialization potential of modified crops (including fruits) because the CRISPR/Cas9 cassette is not inserted into the genome and, in many jurisdictions, the resulting variety is regulated in the same manner as a conventional crop, with certain caveats^21^. CRISPR/Cas9 RNP technology avoids transgene integration by delivering purified RNPs containing the Cas9 protein and gRNA into plant protoplasts and the subsequent regeneration of plants^133,134^. This approach has already been used in apple and grapevine to introduce mutations that confer resistance to fire blight and powdery mildew, respectively^129^. In addition to Cas9 RNPs, CRISPR/Cpf1-RNPs have also been employed successfully for gene editing in protoplasts of soybean and tobacco, paving the way for future use in other crops, including fruits and vegetables^134^. Subsequent optimization experiments permitted plant regeneration from protoplasts and improved the transformation protocol for grape protoplasts, reducing the amount of time needed for RNP delivery and genome editing to less than 3 weeks^131^. It is likely that species- and even cultivar-specific protocol optimization will be necessary to achieve satisfactory editing efficiencies because the major hurdle is not the delivery of RNPs across the protoplast membrane, but the subsequent recovery and regeneration of fertile plants.

### CRISPR base editing

Whereas conventional CRISPR/Cas9 editing tends to introduce short insertions or deletions at the target locus, cytidine and adenosine base editing facilitates the targeted introduction of single nucleotide replacements by direct C-to-T or A-to-G base conversion, respectively. Base editors have been used to introduce herbicide resistance traits in fruit crops in proof-of-concept experiments. For example, CBE in the watermelon *ALS* gene resulted in a single amino acid substitution that was sufficient to confer broad-spectrum and heritable resistance to commercial sulfonylurea herbicides^122^.

### CRISPR flippase

Flp/*FRT* is a yeast site-specific recombinase system in which the recombinase Flp (flippase) catalyzes recombination between two copies of the 34-bp *FRT* site, resulting in the excision or inversion of the intervening DNA, depending on the relative orientation of the *FRT* sites. The Flp/*FRT* system has been used to remove selectable markers in T1 apple, apricot, citrus, and grapevine plants, leaving a single *FRT* site behind as a footprint^2^. These studies laid the foundations for more recent work in which the FLP/FRT system was placed under the control of a heat-shock promoter and incorporated into the CRISPR/Cas9 plasmid, allowing the editing of a disease susceptibility gene in apple and subsequent removal of the CRISPR/Cas9 components^77^. This technology has yet to be applied in other fruit crops, but it shows great promise given the efficiency of editing and T-DNA excision.

### New CRISPR nucleases

Most CRISPR studies thus far have used the endonuclease Cas9 from *Streptococcus pyrogenes* (SpCas9). In its native form, SpCas9 requires a trans-activating CRISPR RNA (tracrRNA) and a CRISPR-RNA (crRNA) to induce blunt double-strand breaks in target DNA. These functions were combined into a single gRNA for the development of CRISPR/Cas9 as an engineering tool. But SpCas9 is only one of a large family of CRISPR-associated nucleases with diverse properties, some of which may be advantageous for genome editing in fruit crops by improving efficiency, specificity, or versatility, or by reducing costs^135^. For example, Cas9 from *Staphylococcus aureus* (SaCas9) differs from SpCas9 in terms of protospacer adjacent motif (PAM) specificity but has a similar editing efficiency. It has been used in several model plant species and also recently in citrus, and provides greater versatility by extending the range of potential genomic targets^126^.

Cas12a/Cpf1 from *Prevotella* and *Francisella* spp. recognizes a T-rich PAM and generates compatible cohesive ends with overhangs of 4–5 nt, differing from the blunt ends introduced by Cas9, and increasing the efficiency of DNA integration (knock-in)^136^. Cas12a/Cpf1 is also a smaller protein than Cas9, which improves the efficiency of multiplex editing. CsmI is also smaller than Cas9^136^, and recognizes AT-rich PAM sites thus improving the accuracy of genome editing in AT-rich regions^135^. This approach has been employed to edit the PDS gene in citrus, establishing the feasibility of Cpf1-mediated, DNA-free editing in fruit crops^137^.

## Conclusions

Genetic engineering facilitates the development of fruits with useful agronomic or quality traits that are difficult or laborious to achieve by conventional breeding, either due to the lack of suitable germplasm or the long breeding cycles and need for multiple rounds of back-crossing. The same traits can be introduced by genetic engineering in one generation, often directly into elite varieties. Some genetically engineered fruits have been on the market for more than 25 years, and have achieved a remarkable positive socioeconomic impact by reducing pests and diseases and increasing the quality of the end product, both of which help to increase income for farmers. Further benefits to farmers, consumers, and the environment reflect the reduced use of pesticides. The development of new molecular breeding technologies such as trans-grafting and genome editing not only offer the promise of further commercial fruit varieties with resistance to biotic and abiotic stresses, improved flavor and nutrient content, and modified flowering and ripening times, but also help to address some of the regulatory constraints that limit the cultivation of first-generation transgenic crops. In particular, the development of transgene-free genome editing methods based on CRISPR/Cas9 and other nucleases offers a way to introduce precise changes at preselected genomic sites with no genetic footprints and no off-targets. In many jurisdictions, some varieties generated through genome editing are exempt from GMO regulations. These tools and techniques are available for the accelerated development of fruit crops with properties that satisfy the needs of producers, retailers, and consumers, in a sustainable and environmentally friendly manner.

## Supplementary information

Table S1

Table S2
