# Supplementary material for: Transgenic and genome-edited fruits: background, constraints, benefits, and commercial opportunities
Source: Hortic Res. 2021 Jul 17;8:166. doi: 10.1038/s41438-021-00601-3 (PMC8286259; doi:10.1038/s41438-021-00601-3)
Supplement: Supplementary file 1 — Table S1 [file 41438_2021_601_MOESM1_ESM.docx]

**Supplementary Information**

**Table S1**

**Transgenic and genome edited fruits: background, constraints, benefits and commercial opportunities**

Maria Lobato-Gómez^1^, Seanna Hewitt^2^, Teresa Capell^1^, Paul Christou^1,3^, Amit Dhingra^2^, Patricia Sarai Girón-Calva^1,*^

^1^Department of Crop and Forest Sciences, University of Lleida-Agrotecnio Center, 25198 Lleida, Spain.

^2^Department of Horticulture, Washington State University, PO Box 646414, Pullman, WA, United States

^3^ICREA, Catalan Institute for Research and Advanced Studies, 08010 Barcelona, Spain

* Corresponding author:

Sarai Girón-Calva ([sarai.giron@udl.cat](mailto:sarai.giron@udl.cat))

Phone: + 34 973 70 25 00

**Table S1**. Engineered fruits approved for commercialization: characteristics. OE, overexpression; GS, gene silencing. Varieties on the market are shown in Table 1.

| Target trait | Species | Trade or event name | Target gene(s) | Encoded product and function | Modification strategy | Outcome |
| --- | --- | --- | --- | --- | --- | --- |
| Quality improvement | Melon  (*Cucumis melo*) | Melon-A and B | *SAM-K* (*Escherichia coli* bactheriophage T) | S-Adenosylmethionine (SAM) hydrolase. Degrades SAM. | OE | Decreased ethylene production results in delayed ripening |
|  | Tomato  (*Solanum lycopersicum*) | 8338 | *ACCD* (*Pseudomonas chlororaphis)* | 1-Amino-cyclopropane-1-carboxylic acid (ACC) deaminase (metabolizes the ethylene precursor ACC) | OE | Decreased ethylene production results in delayed ripening |
|  |  | Endless summer | *acc* (truncated) (*S. lycopersicum)* | The product is a non-functional 1-amino-cyclopropane-1- carboxylic acid (ACC) synthase, which results in decreased production of ACC | GS | Decreased ethylene production results in delayed ripening |
|  |  | 35-1-N | *SAM-K* (*E. coli* bactheriophage T) | S-Adenosylmethionine (SAM) hydrolase which degrades SAM | GS | Decreased ethylene production results in delayed ripening |
|  |  | Huafan No. 1 | *ACO* (anti-efe) (*S. Lycopersicum)* | 1-Amino-cyclopropane-1- carboxylate oxidase (catalyzes the formation of ethylene from 1-amino-cyclopropane-1- carboxylic acid) | GS | Decreased ethylene production results in delayed ripening |
|  |  | FLAVR SAVR, Da, B and F ((1401F, h38F, 11013F, 7913F) | *PG* (sense or antisense) (*S. lycopersicum)* | Polygalacturonase (hydrolytic enzyme which degrades pectin) | GS | Decreased pectin degradation in the cell wall results in delayed fruit softening |
|  | Apple  (*Malus x Domestica*) | Arctic® Apple Golden Delicious (GD743), Granny Smith (GS784), Fuji (NF872) | *PGAS ppo* (*M. domestica)* | Polyphenol oxidase (PPO) (catalyzes the oxidation of phenols to quinones) | GS | Decreased phenol oxidation eliminates browning |
| Nutritional improvement | Pineapple  (*Ananas comosus*) | Pinkglow™ (EF2-114) | *PSY* (*Citrus unshiu)*  *b-Lyc* *(A. comosus)*  *e-Lyc* (*A. comusus)* | Phytoene synthase (PSY) catalyzes the formation of phytoene, a precursor of lycopene. The β-cyclase and ε-cyclase enzymes convert lycopene to γ-carotene and δ-carotene, respectively. | OE | Increased production of carotenoids results in a fruit with pink color |
| Disease resistance | Papaya  (*Carica papaya* L.) | Rainbow and SunUp (55-1), 63-1 and X17-2 | *PRSVcp* (Papaya ringspot virus) | Coat protein of the PRSV strain HA 5-1 and H1-K | OE | Expression of the coat protein confers resistance to PRSV through a pathogen-derived resistance mechanism |
|  |  | Huanoning No. 1 | No information | No information | OE | No information |
|  | Sweet pepper  (*Capsicum annuum*) | PK-SP01 | *CMVcp* (Cucumber mosaic virus) | Encodes the coat protein of the CMV | OE | Expression of the coat protein confers resistance to CMV through a pathogen-derived resistance mechanism |
|  | Plum  (*Prunus domestica*) | C-5 | *PPVcp* (Plum pox virus) | Encodes the coat protein of the PPV | OE | Expression of the coat protein confers resistance to PPV through a pathogen-derived resistance mechanism |
|  | Tomato (*Solanum lycopersicum*) | 8805R | *CMVcp* (Cucumber mosaic virus) | Coat protein of the CMV which confers resistance | OE | Expression of the coat protein confers resistance to CMV through a pathogen-derived resistance mechanism |
|  | Squash  (*Cucurbita pepo*) | ZW20 and CZW3 | *CMVcp* (Cucumber mosaic virus), *ZYMVcp* (Zucchini yellow mosaic virus), *WMV2cp* (Watermelon mosaic virus2) | Coat proteins of the CMV, ZYMV and WMV2 | OE | Expression of the coat proteins confers resistance to CMV, ZYMV and WMV2 through a pathogen-derived resistance mechanism |
| Pest resistance | Eggplant  (*Solanum melongena* L.) | Bari Bt Begun 1, 2, 3, and 4 | *Cry1Ac* (*Bacillus thuringiensis)* | Cry1Ac insecticidal protein | OE | Expression of the Cry1Ac protein confers resistance to fruit and shoot borer (FSB),*Leucinodes orbonalis* |
|  | Tomato (*Solanum lycopersicum*) | 5345 | *Cry1Ac* (*B. thuringiensis* subs. Kurstaki strain HD73) | Cry1Ac insecticidal protein | OE | Expression of the Cry1Ac protein confers resistance to lepidopteran insects |
