# Supplementary material for: Transgenic and genome-edited fruits: background, constraints, benefits, and commercial opportunities
Source: Hortic Res. 2021 Jul 17;8:166. doi: 10.1038/s41438-021-00601-3 (PMC8286259; doi:10.1038/s41438-021-00601-3)
Supplement: Supplementary file 2 — Table S2 [file 41438_2021_601_MOESM2_ESM.pdf]

Supplementary Information

Table S2

Transgenic and genome edited fruits: background, constraints, benefits and commercial opportunities

Maria Lobato-Gómez[1], Seanna Hewitt[2], Teresa Capell[1], Paul Christou[1,3], Amit Dhingra[2], Patricia Sarai Girón-Calva[1,*]

[1]Department of Crop and Forest Sciences, University of Lleida-Agrotecnio Center, 25198 Lleida, Spain.

[2]Department of Horticulture, Washington State University, PO Box 646414, Pullman, WA, United States

[3]ICREA, Catalan Institute for Research and Advanced Studies, 08010 Barcelona, Spain

* Corresponding author:

Sarai Girón-Calva (sarai.giron@udl.cat)

Phone: + 34 973 70 25 00

Table S2. Fruit and fruit trees under development through genetic engineering and genome editing with potential socio-economic impact (until mid-2020). Gene delivery was through *Agrobacterium tumefaciens*-mediated transformation unless indicated otherwise.

Species	Cultivar	Target trait	Target gene(s) and source	Encoded product and function	Modification strategy	Outcome	Type of study	Ref.
Tomato (*Solanum lycopersicum*)	'MP-1'	Parthenocarpy	*AGL6*	Transcription factor regulator of parthenocarpy	CRISPR/Cas9	100% of fruits of WT plants had more than 10 seeds, whereas 78-85% mutants had 0 seeds/fruit, and 5% mutants had less than 10 seed/fruit	Field trials	(Klap *et al.*, 2017)
Apple (*Malus* x *domestica)*	'Galaxy' and 'M26'	Increased resistance to *Erwinia amylovora, Venturia inaequalis* and *Gymnosporangium juniperi-virginianae*	*NPR1-1*	Protein regulating the expression of pathogenesis-related genes	Transgenesis (Overexpression)	Transformed lines had only 32-40% of shoot length infected, compared with 80% in control plants.	Field trials	(Malnoy *et al.*, 2007)
Banana *(Musa* spp.)	'Sukali Ndiizi' and 'Mpologoma'.	Increased resistance to *Xanthomonas campestris pv. musacearum*	*Harp* from *Capsicum annuum*	Hypersensitive response assisting protein	Transgenesis (Overexpression)	No wilted leaves were observed in transgenic plants, most leaves in WT plants were wilted	Greenhouse, Field trials	(Tripathi *et al.*, 2010, 2014)
Banana *(Musa* spp.)	'Sukali Ndiizi' and 'Mpologoma'.	Increased resistance to *X. campestris pv. musacearum*	*Pflp* from *C. annuum*	Ferredoxin-like protein which increases resistance against bacterial pathogens by intensifying the production of active oxygen species and activation of the hypersensitive response	Transgenesis (Overexpression)	Transgenic lines evaluated did not have wilted leaves whereas control plants showed complete wilting	Laboratory, Greenhouse, and Field trials	(Namukwaya *et al.*, 2012; Tripathi *et al.*, 2014)
Papaya (*Carica papaya*)	'Maradol'	Fruit ripening	ACC oxidase	Enzyme involved in fruit ripening	Transgenesis (RNA silencing)	Fruits did not ripen after 21 days on the tree, whereas WT did before 15 days on tree.	Field trials	(López-Gómez *et al.*, 2009)
Grapevine (*Vitis vinifera*)	'41B', 'SO4', and 'Chardonnay'	Resistance to Grapevine fanleaf virus (GFLV)	CP of GFLV	Coat protein (CP) of GFLV	Transgenesis (overexpression)	Three transgenic lines had 0% GFLV infection compared with 70% in the WT plants	Field trials	(Mauro *et al.*, 1995; Vigne, Komar and Fuchs, 2004)
Tomato (*Solanum lycopersicum*)	'M82' and 'Sweet 100'	Harvest time	*SP5G*	SELF-PRUNING 5G is a flowering repressor	CRISPR/Cas9	Harvest time was reduced two weeks in *SP5G*-mutants compared to WT plants	Field trials	(Soyk *et al.*, 2017)
Tomato (*Solanum lycopersicum*)	'M82'	Shelf life	*ALC*	Alcobaca *is a* natural occurring mutation related to long-shelf life	CRISPR/Cas9	Fruits of *ALC*-mutants had increased shelf-life (40 days) compared to fruits from WT plants	Field trials	(Yu *et al.*, 2017)
Apple (*Malus* x *domestica)*	'Pinova'	Floral initiation	*MADS4* from *Betula pendula* Roth.	Regulator of floral meristem identity	Transgenesis (Overexpression)	Flowering occurred within 3-4 months compared to ~2 years for WT trees	Greenhouse	(Flachowsky *et al.*, 2007)
Apple (*Malus* x *domestica)*	'Pinova'	Increased resistance to *E. amylovora*	*Dpo* from *E. amylovora* phage phi-Ea1h	Depolimerase that binds to the capsular extracellular polysaccharide (EPS) of *E. amylovora* and degrades the bacterial EPS polysaccharide	Transgenesis (Overexpression)	Reduced susceptibility to *E. amylovora* was observed for some clones (23% decrease relative to WT)	Greenhouse	(Flachowsky *et al.*, 2008)

Apple (*Malus pumila)*	'Greensleeves' clone GS92	Increased resistance to drought and cold stress	*MYB4* from *Oriza sativa*	Transcription factor regulating the expression of genes involved in biotic and abiotic stress responses	Transgenesis (Overexpression)	After 15 days of drought the relative water content decreased 15% in WT but did not decrease in transformed plants. Glucose, fructose, sucrose content was 35%, 30% and 38% higher in transgenic plants relative to WT. Proline content was 38% higher in transgenic plants relative to WT after 2 days of cold stress	Greenhouse	(Pasquali *et al.*, 2008)
Apple (*Malus* x domestica	'A2', 'Gravenstein' and 'McIntosh'	Decreased plant size	*GAI* from *Arabidopsis thaliana*	Protein regulating plant responses to gibberellins	Transgenesis (Overexpression)	Stem length was reduced 79% for 'McIntosh', 75% for 'Gravenstein' and 47% for 'A2' relative to their WT plants	Greenhouse	(Zhu, Li and Welander, 2008)
Apple (*Malus* x *domestica*	'Gala' and 'Elstar'	Increased resistance to *V. inaequalis*	*HcrVf1* from *M. floribunda* 821	Receptor-like protein which forms a gene-for-gene relationship with races of *V. inaequalis*	Transgenesis (Overexpression)	Of the 6 resistant lines obtained, 4 lines had no macroscopically visible symptoms, 1 line had hypersensitive pinpoints and 1 line had chlorotic and necrotic areas with no sporulation	Greenhouse	(Szankowski, Waidmann, *et al.*, 2009)
Apple (*Malus* x *domestica*	'Pinova'	Flowering	*FT1*	Flowering integrator	Transgenesis (Overexpression)	Flowering was accelerated 2-3 weeks from flowering induction to full bloom relative to 45-48 weeks in non-transgenic apple	Greenhouse	(Tränkner *et al.*, 2010)
Apple (*Malus* x *domestica*	'GL-3'	Plant size	*NAC1*	Transcription factor regulating plant size	Transgenesis (Overexpression)	Transgenic plants were 31% shorter than the WT	Greenhouse	(Jia *et al.*, 2018)
Apple (*Malus* x *domestica*	'Bolero'	Fruit shape	*PI*	Regulator of petal and stamen identity	Transgenesis (Overexpression)	Plants produced flattened fruit because of reduced cell growth (66%) at the basipetal position of the fruit	Greenhouse	(Yao *et al.*, 2018)
Apple (*Malus* x *domestica*	'Fuji Raku Raku'	Increased carotenoid content	*DRX from A. thaliana*	Enzyme involved in the formation of methyl erythritol phosphate, the first precursor for plastid isoprenoids	Transgenesis (Overexpression)	Transgenic seedlings displayed up to 3-fold increase in total carotenoids compared to WT, whereas transient transformation of fruits induced a 2-fold increment in total carotenoids	Greenhouse	(Arcos *et al.*, 2020)
Banana *(Musa acuminata*)	'Rasthali'	Increased resistance to Banana bunchy top virus	*ihpRNA-Rep* and *ighRNA-ProRep*	Viral master replication initiation proteins	Transgenesis (Gene silencing)	Absence of disease symptoms in transgenic plants after 6 months of viruliferous aphid inoculation	Greenhouse	(Shekhawat, Ganapathi and Hadapad, 2012)
Banana (*Musa balbisiana*)	'Gonja Manjaya'	Silencing the latency of the Banana streak virus (BSV) in the host genome	*eBSV*	Endogenous BSV integrated in the host genome	Targeted mutagenesis (CRISPR/Cas9)	Broken or continuous streaks of yellow, chlorotic, black, or brown color on leaves appeared in all WT control plants, whereas	Greenhouse	(Tripathi *et al.*, 2019)

						75% of edited plants remained asymptomatic		
Banana (*Musa spp*.)	'Gros Michel'	Plant size	*MAGA20ox2*	Gene regulating gibberellin (GA) production	Targeted mutagenesis (CRISPR/Cas9)	The height of edited plants was reduced 75% relative to WT	Greenhouse	(Shao *et al.*, 2020)
Blueberry *(Vaccinum corymbosum)*	'Aurora'	Flower development/Yield improvement	*SOC1K*	Flowering integrator which also regulates plant growth and development	Transgenesis (Overexpression)	Number of floral buds in transgenic plants increase by 150% compared to chilled WT	Greenhouse	(Song and Chen, 2018)
Blueberry *(Vaccinum corymbosum)*	'Legacy'	Floral initiation	*FLC3-like* from *M. domestica*	Transcription factor that represses flowering genes	Transgenesis (Overexpression)	WT plants did not flower under non-chilling conditions, whereas transgenic plants had 37 flower clusters	Greenhouse	(Zong *et al.*, 2019)
Cherry (*Prunus avium*)	'Mazzard' and 'Longguan'	Fruit size	*CYP78A9*	Regulator of fruit size and organ development	Transgenesis (Gene silencing)	Fruit longitudinal diameter was reduced 56% whereas horizontal diameter was decreased 63% compared to WT	Greenhouse	(Qi *et al.*, 2017)
Mexican lime (Citrus aurantifolia)	N.A.	Increased resistance to Citrus tristeza virus (CTV)	*p25CP*	Coat protein of the CTV	Transgenesis (Overexpression)	25-33% transgenic lines did not develop symptoms, whereas all control plants became infected	Greenhouse	(Domínguez *et al.*, 2002)
Trifoliate orange (*Poncirus trifoliata)*	N.A.	Floral initiation	*CiFT*	Floral integrator	Transgenesis (Overexpression)	Transgenic trees started to flower as early as 12 weeks after transfer to a greenhouse, whereas WT plants had longer (6-20 year) juvenile periods	Greenhouse	(Endo *et al.*, 2005)
Carrizo citrange (*Citrus sinensis* x *Poncirus trifoliata)*	N.A.	Plant stature	*GA20ox1* sense and antisense	Enzyme which catalyzes the conversion of the gibberellins GA12/GA53, to GA9/GA20	Transgenesis (Overexpression and gene silencing)	Branch length was longer in sense (120%) and shorter (50%) in antisense plants	Greenhouse	(Fagoaga *et al.*, 2007)
Trifoliate orange (*Poncirus trifoliata)*	N.A.	Parthenocarpy	*MAC12.2* from *A. thaliana*	Induces parthenocarpy	Transgenesis (Overexpression)	Seed number decreased 83% in transgenic plants relative to WT	Greenhouse	(Tan *et al.*, 2009)
Mexican lime (*Citrus aurantifolia*)	N.A.	Increased resistance to Citrus tristeza virus	*3DF1* and *3CA5*	Monoclonal antibodies against CTV p25 major coat protein	Transgenesis (Overexpression)	Transgenic plants had 40%-60% of virus propagations, whereas WGT plants showed 95% of virus propagation	Greenhouse	(Cervera *et al.*, 2010)
Mexican lime (*Citrus aurantifolia*)	N.A.	Increased resistance to Citrus tristeza virus (CTV)	*3'p23+3'UTR* intron harpin from CTV	RNA-binding protein involved in regulating the balance of plus and minus RNA strands during replication	Transgenesis (Overexpression)	9%–56% of the propagations remaining uninfected, whereas all WT were symptomatic	Greenhouse	(Lopez *et al.*, 2010)
Citrumelo (*Citrus paradisi* 'Duncan' × *Poncirus trifoliata*)	'Swingle'	Increased tolerance to drought stress	*P5CSF129A* gene from *V. aconitifolia*	Key enzyme for proline biosynthesis	Transgenesis (Overexpression)	Proline content was 2.5-fold higher in transgenic plants than control plants	Greenhouse	(de Campos *et al.*, 2011)
Sweet orange (*Citrus sinensis*)	'Navelina' and 'Pineapple'	Increased resistance to *X. citri* subsp. *Citri* and *Penicillium digitatum*	*MTSE1* from *Citrus unshiu*	Limonene synthase which catalyzes the formation of limonene	Transgenesis (Downregulation)	The production of limonene decreased 85 times in transgenic fruits relative to control plants. The percentage of infected wounds with *P. digitatum*	Greenhouse	(Rodríguez *et al.*, 2011)

						was 18.5% in transgenic fruits compared to 60% in control fruits. *X. citri* sp. *citri*, infected wounds in transgenic fruits was <10% compared to 65.7% in control fruits		
Sweet orange (*Citrus sinensis*)	'Succari' and 'Bingtang'	Increased resistance to *X. axonopodis* pv. *citr*i	*pthA-nls*	Pathogenesis gene encoding 3 nuclear localization signals that are critical for PthA's function and localization to the host cell nucleus	Transgenesis (Overexpression)	Disease incidence of 23.3% for 'Sucarri' sweet orange and 33.3% for 'Bingtang', whereas control plants had 100% of disease incidence	Greenhouse	(Yang *et al.*, 2011)
Grapefruit (*Citrus paradisi*)	'Duncan'	Increased resistance to *Elsinoë fawcettii*	*attE* from *Hyalophora cecropia*	Attacin E is an antimicrobial peptide that increases membrane permeability in Gram negative bacteria	Transgenesis (Overexpression)	Control plants had >10 postules on each leaf, whereas transgenic plants had 4-6 postules per leaf	Greenhouse	(Mondal *et al.*, 2012)
Sweet orange (*Citrus sinensis*)	'Pineapple'	Increased carotenoid content	*Csb-CHX*	β-carotene hydroxylase catalyzing the conversion of β-carotene to xanthophylls.	Transgenesis (Gene silencing)	26-fold increase of β-carotene in the pulp of transgenic fruits relative to WT	Greenhouse	(Pons *et al.*, 2014)
Sweet orange (*Citrus sinensis*)	'Hamlin' and 'Valencia'	Increased resistance to *Candidatus Liberibacter asiaticus* (CLas)	*NPR1* from A. thaliana	Regulator of transcription factors controlling pathogen-related gene expression and mediates the salicylic acid induced responses	Transgenesis (Overexpression)	Control trees tested positive for the presence of CLas within 6 months after infection, whereas 46% of transgenic plants tested negative	Greenhouse	(Dutt *et al.*, 2015)
Carrizo citrange (*Citrus sinensis* x *Poncirus trifoliata)*	N.A.	Increased resistance to *X. citri subsp. Citri*	*Mthionin*	Modified thionin, cysteine-rich peptide which shows antibacterial activity	Transgenesis (Overexpression)	Bacterial population in transgenic plants was <1E+5 log whereas in control plants was >1E+8 log	Greenhouse	(Hao, Stover and Gupta, 2016)
Blood orange (*Citrus sinensis* Osbeck)	'Tarocco'	Increased resistance to *Candidatus Liberibacter asiaticus* (CLas)	*CB, PR1aCB* and *PR1aCBer* from *Antheraea pernyi*	Cecropin B, antimicrobial cationic lytic peptide	Transgenesis (Overexpression)	Bacterial populations were not detected in transgenic lines, whereas bacterial population were detected in WT (31.2 vs. 16 Ct for bacterial 16S gene, respectively)	Greenhouse	(Zou *et al.*, 2017)
Blood orange (*Citrus sinensis* Osbeck)	'Wanjincheng'	Increased resistance to *X. citri subsp. Citri*	*WRKY22*	Transcription factor involved in the regulation of plant responses to biotic stress	Targeted mutagenesis (CRISPR/Cas9)	Smaller diseased areas were found in transgenic plants (0.80 mm^2), compared with the WT (1.60 mm^2)	Greenhouse	(L. Wang *et al.*, 2019)
Grapevine (*Vitis berlandieri* x *Vitis rupestris*)	'Richter'	Tolerance to salinity and oxidative stress	*MsFer* from *Medicago sativa*	Ferritin iron-storing protein	Transgenesis (overexpression)	Transgenic plants showed 30% lower loss of photosynthetic activity than control plants under salinity and oxidative (paraquat) stress	Greenhouse	(Zok *et al.*, 2010)
Kiwifruit (*Actinidia deliciosa*)	'Hayward'	Increased carotenoid content	*GGPS, PDS, ZDS, CHX,* and *PSY* from *Citrus unshiu*	Geranylgeranyl diphosphate synthase, phytoene desaturase, ζ-carotene desaturase, β-carotene hydroxylase, and phytoene synthase.	Transgenesis (overexpression)	Lutein and β-carotene content increased 1.2 and 1.3-fold, respectively, in transgenic plants compared to control plants	Greenhouse	(Kim *et al.*, 2010)

				Enzymes involved in carotenoid biosynthesis				
Ks	'Qin mei'	Increase tolerance to salinity stress	*NHX1* from *A. thaliana*	Vacuolar Na^+/H^+ antiporter	Transgenesis (overexpression)	Transgenic kiwifruit plants had 2 times more leaf Na^+ and K^+ under 200 mmol/l NaCl relative to WT	Greenhouse	(Tian, Wang and Xu, 2011)
Melon (*Cucumis melo*)	'Silver Light'	Increased resistance to Papaya ringspot virus (PRSV W) and Zucchini yellow mosaic virus (ZYMV)	Partial *CP* of PRSV W and ZYMV	Partial coat proteins of PRSV W and ZYMV	Transgenesis (overexpression)	Transgenic plants did no exhibit any symptom after the inoculation with ZYMV and PRSV W, whereas 51% to 84% control plants showed symptoms	Greenhouse	(Wu *et al.*, 2010)
Pear (*Pyrus communis*)	'Passe Crassane'	Increased resistance to *E. amylovora*	Bovine *Lactoferrin*	Iron chelator protein with bactericidal action	Transgenesis (overexpression)	Symptoms of fire blight infection decreased up to 60% in transgenic plants compared to control plants	Greenhouse	(Malnoy *et al.*, 2003)
Pear (*Pyrus communis*)	'La France'	Ethylene production	*ACO*	1-aminocyclopropane-1-carboxylic acid oxidase involved in ethylene biosynthesis	Transgenesis (overexpression and gene silencing)	*ACO* (antisense) mutants had decreased ethylene production of up to 85% relative to control shoots, whereas *ACO* overexpression increased ethylene production 200%	Greenhouse	(Gao *et al.*, 2007)
Pear (*Pyrus communis*)	'Spadona'	Floral initiation	*TFL1-1* and *TFL1-2*	Floral repressor	Transgenesis (gene silencing)	Flowers in a transgenic line developed 1-8 months after transfer to greenhouse in a transgenic line, whereas control plants did not flower	Greenhouse	(Freiman *et al.*, 2012)
Pear (*Pyrus communis*)	'Old Home'	Tocopherol production	*VTE1* from *Juglans regia*	Tocopherol cyclase VTE1 is a key enzyme involved in tocopherol production	Transgenesis (overexpression)	Transgenic pear plants accumulated tocopherol 2.3-fold higher in roots, and 16.2-fold higher in stem tissues relative to WT	Greenhouse	(Wang *et al.*, 2015)
Chili pepper (*Capsicum annum*)	'Aiswarya 2103'	Increased tolerance to salinity stress	Osmotin from *Nicotiana tabacum*	Pathogenesis-related protein induced under abiotic stress	Transgenesis (overexpression)	Under salinity stress, control plants produced 2.2 kg of chili pepper fruits per plant, whereas transgenic plants produced 3.3 kg of chili pepper fruits per plant	Greenhouse	(Subramanyam *et al.*, 2011)
Chili pepper (*Capsicum annum*)	'Nokkwang'	Increased resistance to *Colletotrichum gloeosporioides*	*J1-1*	Antimicrobial peptide (defensin) that inhibits the growth of of a broad range of phytopathogenic fungi	Transgenesis (overexpression)	Transgenic fruits exhibited 25% less anthracnose lesions compared to control plants	Greenhouse	(Seo *et al.*, 2014)
Chili pepper (*Capsicum annum*)	'G4'	Increased tolerance to salinity stress	*NHX2* from *Triticum aestivum*	Na^+/Ha^+ antiporter	Transgenesis (overexpression)	Transgenic plants exhibited 13% less ion leakage than WT plants under 100mM of NaCl	Greenhouse	(Bulle, Yarra and Abbagani, 2016)
Chili pepper (*Capsicum annum*)	'Nokkang'	Increased resistance to *C. letotrichum gloeosporioides*	*EST*	Esterase enzyme that acts on components of the fungus and/or plant cell	Transgenesis (overexpression)	Transgenic fruits exhibited 35% less disease rate than control plants	Greenhouse	(Ko *et al.*, 2016)
Plum (*Prunus domestica*)	N.A.	Chilling requirement	*FT1* from *Populus trichocarpa*	Flowering Locus T1 coordinates vegetative and reproductive growth	Transgenesis (overexpression)	Transgenic plants flowered and produced fruits in greenhouse within 1 to 10 months. Plants did not enter dormancy after cold	Greenhouse	(Srinivasan *et al.*, 2012)

						treatment		
Plum (*Prunus domestica*)	'Startovaya'	Increased resistance to Plum pox virus (PPV)	Hairpin of *PPV-CP*	PPV coat protein derived hairpin construct	Transgenesis (gene silencing)	Transgenic trees were used as rootstock. Grafted trees remained uninfected over 9 years	Greenhouse	(Sidorova *et al.*, 2019)
Strawberry (*Fragaria* × *ananassa*)	'Calypso'	Flavonoid content	*CHS* (antisense)	Chalcone synthase, involved in flavonoid biosynthesis	Transgenesis (gene silencing)	Flavonoid content decreased 50% in transgenic lines compared with control plants	Greenhouse	(Lunkenbein *et al.*, 2006)
Strawberry (*Fragaria* × *ananassa*)	'Anther'	Sugar composition and content	*AGPase FagpS*	ADP-glucose pyrophosphorylase small subunit involved in starch biosynthesis	Transgenesis (gene silencing)	Starch content decrease 27-47% in transgenic plants relative to control plants, whereas soluble sugars increased 16-37%	Greenhouse	(Park *et al.*, 2006)
Strawberry (*Fragaria* × *ananassa*)	'Anther'	Fruit softening	*Cel1*	Endo-β-(1,4)-glucanase involved in fruit cell wall degradation during fruit ripening	Transgenesis (overexpression and gene silencing)	Firmness increased 16-36% and 22-25% in transgenic plants (antisense) (at the turning and red stage, respectively) relative to control plants	Greenhouse	(Lee and Kim, 2011)
Wild straweberry (*Fragaria vesca*)	'Ruegen'	Phosphorous content	*miR399*	MicroRNA399 involved in the regulation of phosphate homeostasis	Transgenesis (overexpression)	Phosphorous content increased 1.1-fold to 2.1-fold in leaves and fruits of transgenic plants relative to WT plants	Greenhouse	(Y. Wang *et al.*, 2017)
Strawberry (*Fragaria* × *ananassa*)	'Reine des Vellés'	Flower development	*TM6*	Transcription factor regulating flower development	CRISPR/Cas9	Transgenic plants had 75% less pollen grains than WT plants	Greenhouse	(Martín-Pizarro, Triviño and Posé, 2019)
Tomato (*Solanum lycopersicum*)	'VF36'	Increased resistance to *X. campestris* (*Xcv*)	*Bs2* from *Capsicum annuum*	Member of the nucleotide binding site–leucine-rich repeat (NBS-LRR) class of R genes. Recognizes and confers resistance to *Xanthomonas*	Transgenesis (overexpression)	Transgenic plants lack chlorotic disease symptoms on leaves inoculated with Xcv (visual evaluation)	Greenhouse	(Tai *et al.*, 1999)
Tomato (*Solanum lycopersicum*)	'Money Maker', 'Rio Grande–PtoS', and 'Rio Grande–PtoR'	Increased resistance to *Pseudomonas syringae*	*Pto*	Serine/threonine protein kinase involved in the activation of the hypersensitive response against bacterial pathogens	Transgenesis (overexpression)	Bacterial number was 10-fold lower in 48-2 than in Money Maker s, and 10- to 50-fold lower in 11-12 and 13-8 than in PtoR and PtoS	Greenhouse	(Tang *et al.*, 1999)
Tomato (*Solanum lycopersicum*)	'Micro-Tom'	Increased tolerance to drought stress	*PlP1;3* from *Malus domestica*	Aquaporin, a water channel	Transgenesis (overexpression)	Transgenic lines had 20% more mass than WT after 20 days of drought	Greenhouse	(L. Wang *et al.*, 2017)
Tomato (*Solanum lycopersicum*)	'FL8000'	Increased resistance to *P. syringae* and *X. perforans*	*DMR6*	Downy mildew resistance 6 is associated with salicylic acid homeostasis	CRISPR/Cas9	*DMR6*-mutants had lower levels of *P. syringae* (5.8 log CFU/ cm^2) and X. perforans (4.2 log CFU/ cm^2) compared to WT plants (7 and 6 log CFU/ cm^2, respectively)	Greenhouse	(de Toledo Thomazella *et al.*, 2016)
Tomato (*Solanum lycopersicum*)	N.A.	Fruit ripening	*ORRM4*	ORRM proteins regulates plant growth, flowering time, and fruit ripening	CRISPR/Cas9	Fruit ripening was delayed 10 days in *ORRM*-mutants compared to WT fruits	Greenhouse	(Yang *et al.*, 2017)
Tomato (*Solanum lycopersicum*)	'AC'	Lycopene content	*SGR1, LCY-E, Blc, LCY-B1*, and *LCY-B2*	Key enzymes of lycopene and β- and α-carotene biosynthesis	CRISPR/Cas9	Lycopene content increased 5.1-fold in fruits from mutants plants	Greenhouse	(X. Li *et al.*, 2018)

						compared to control fruits		
Tomato (*Solanum lycopersicum*)	'LA1589', 'LA1357', 'LA1547', and 'LA1606'	Inflorescence, Fruit size and Vitamin C content	*SP5G, SP, CLV3, WUS,* and *GGP1*	Key regulators of flowering and fruit locule number, key enzyme involved in vitamin C biosynthesis	CRISPR/Cas9	In edited plants, the first inflorescence appeared after 7 leaves (compared to 12 leaves in WT), 5–12% of fruits had three locules, and vitamin C decreased 48% relative to WT plants	Greenhouse	(T. Li *et al.*, 2018)
Tomato (*Solanum lycopersicum*)	'Ailsa Craig' and 'Micro-Tom'	γ-Aminobutyric acid (GABA) content	*TP1, TP2, TP3, CAT9,* and *SSADH*	Key enzymes involved GABA metabolism	CRISPR/Cas9	GABA content increased 19-fold in mutant plants compared with WT plants	Greenhouse	(R. Li *et al.*, 2018)
Wild tomato (*Solanum pimpinellifolium*)	N.A.	Plant morphology, fruit number and size, lycopene content	*SP, O, FW 2.2, CycB, MULT,* and *FAS*	Key regulators of flowering, fruit shape, fruit size, fruit number, key enzyme involved in the conversion of lycopene into β-carotene	CRISPR/Cas9	Plant height was reduced 50%, inflorescences were increased 500%, locule number was increase 350%, and lycopene accumulation was 500% higher in edited plants than WT plants	Greenhouse	(Zsögön *et al.*, 2018)
Tomato (*Solanum lycopersicum*)	'Moneyberg', 'Ailsa Craig', and 'AC'	Fruit development and ripening	*AP2a, NAC-NOR, FUL1,* and *FUL2*	Key regulators of ethylene production and fruit ripening	CRISPR/Cas9	AP2a mutants exhibited an earlier ripening, fruits reached the Breaker stage in 39-41 days, whereas in the wild-type was reached in 47 days. Pericarp of AP2a mutants remained orange/brown, compared with the fully red of the wild-type. NOR mutants ripened 3 days later than wild-type fruits.	Greenhouse	(R. Wang **et al.**, 2019)
Watermelon (*Citrullus lanatus*)	'ZG94'	Increased herbicide resistance	*ALS*	Key enzyme in branched amino acid biosynthesis	CRISPR/Cas9	ALS homozygous mutants were not affected after 14 days of a treatment with tribenuron (a broad weed controlling herbicide), whereas WT plants were severely damaged (visual evaluation)	Greenhouse	(Yu and Powles, 2014; Tian *et al.*, 2018)
Walnut (*Juglans regia*)	'Sunland'	Increased resistance to *Cydia pomonella*	*cryIA* **from** *Bacillus thuringiensis*	Insecticidal crystal protein fragments (1CPFs)	Transgenesis (gene silencing)	Codling moth mortality was 80-100% in transgenic walnuts versus 9.2% in WT	Greenhouse	(Dandekar *et al.*, 1998)
Watermelon (*Citrullus lanatus*)	Feeling', 'China Baby', and 'Quality'	Resistance to Zucchini yellow mosaic virus (ZYMV) and Papaya ringspot virus type W (PRSV W)	Truncated ZYMV and PRSVW *CP*	Coat protein, it confers resistance to ZYMV and PRSV W	Transgenesis (gene silencing)	A transgenic line exhibited100% resistance to ZYMV and PRSV W after 4 weeks of virus inoculation (visual evaluation) and all plants from this line were virus-negative by Western blot	Greenhouse	(Yu *et al.*, 2011)
Watermelon (*Citrullus lanatus*)	'Feeling'	Increased resistance to Watermelon silver mottle virus (WSMoV), Cucumber mosaic virus (CMV), Cucumber green	partial *N* gene from WSMoV, and partial *CP* from CMV, CGMMV, and WMV	Nucleocapsid (N) and coat protein (CP) confer resistant to WSMoV, CMV, CGMMV, and WMV	Transgenesis (gene silencing)	In two transgenic lines, 100% of the plants did not show symptoms during 5 weeks of observation after the inoculation of the virus individually and collectively. Viruses were not detected	Greenhouse	(Lin *et al.*, 2012)

		mottle mosaic virus (CGMMV), and Watermelon mosaic virus (WMV)				by ELISA in these two lines.		
Pear (*Pyrus communis*)	'Duli'	Plant size	*PAT14*	S-acyltransferase, involved in stem and leaf development via endogenous hormone signaling	CRISPR/Cas9 targeted mutagenesis	Transgenic plants were 67% smaller than WT plants	Laboratory	(Pang *et al.*, 2019)
Pummelo (*Citrus grandis*)	N.A.	Increased tolerance to cold stress	*PtrbHLH* from *Poncirus trifoliata*	Transcription factor regulating plant responses to abiotic stress	Transgenesis (Overexpression)	Electrolyte leakage in transgenic plants was 13% lower compared to WT (25%). Proline content was higher in transgenic plants (210-250 µg/g) compare WT (150 µg/g)	Laboratory	(Geng *et al.*, 2019)
Grapefruit (*Citrus paradisi*)	'Duncan'	Improved palatability by altering flavonoid accumulation	*CHS* and *CHI* sense and antisense	Chalcone synthase and chalcone isomerase catalyze the formation of naringenin chalcone and its conversion to naringenin flavanone	Transgenesis (Overexpression and gene silencing)	Transgenic plants had 28% less total flavonoid content than WT	Laboratory	(Koca *et al.*, 2009)
Wild strawberry (*Fragaria vesca*)	'Hawaii 4'	Auxin content	*YUC10*	Protein involved in auxin biosynthesis	CRISPR/Cas9	Free auxin decreased 40% in young fruit of *YUC10*-mutants compared with WT	Laboratory	(Feng *et al.*, 2019)
Tomato (*Solanum lycopersicum*)	'Money Maker'	Increased resistance to powdery mildew (*Oidium neolycopersici*)	*Mlo1*	Negative regulator of powdery mildew resistance	CRISPR/Cas9	Leaves of *Mlo*-mutants did not shown chlorotic spots 5 weeks post inoculation (visual evaluation)	Laboratory	(Nekrasov *et al.*, 2017)
Kiwifruit (*Actinidia deliciosa*)	N.A.	Delayed ripening	*ACO* from *Actinidia deliciosa*	1-aminocyclopropane-1-carboxylic acid oxidase involved in ethylene biosynthesis	Transgenesis (overexpression)	Ethylene production was totally inhibited in transgenic plants whereas control plants produced 1 µmol/kg s	Laboratory	(Atkinson *et al.*, 2011)
Apple (*Malus* x *domestica*)	'M36'	Increased rooting capacity	*rolB* from *Agrobacterium tumefaciens*	Induces adventitious root formation in woody plants	Transgenesis (Overexpression)	Stem discs of transformed clones developed more roots (10 vs. 2) and had higher rooting capacity (>70% vs. 10%) than control plants	Laboratory	(Welander *et al.*, 1998)
Apple (*Malus* x *domestica)*	'M9/29'	Increased rooting capacity	*rolB* from *A Agrobacterium tumefaciens*	Induces adventitious root formation in woody plants	Transgenesis (Overexpression)	The root number in clones increased (3-9) compared to control plants (1). 83-100% of the tested clones rooted whereas only 1% of control plants did	Laboratory	(Zhu *et al.*, 2001)
Apple (*Malus* x *domestica)*	'Greensleeves'	Sorbitol production	*A6PR*	Aldose-6-P reductase that catalyzes the conversion of glucose-6-P into sorbitol-6-P	Transgenesis (Gene silencing)	Sorbitol content was reduced 66% relative to WT. Sucrose and starch content increased (150% and 87%, respectively) in mature leaves	Laboratory	(Cheng *et al.*, 2005)
Apple (*Malus* x *domestica)*	'Elstar E94'	Decreased allergen content	*Mald1*	Apple allergen	Transgenesis (Gene silencing)	Reactivity (SPT/ histamine ratio) decreased 70%	Laboratory	(Gilissen *et al.*, 2005)
Apple	'Royal Gala'	Fruit color	*MYB10* from *M.*	Transcription factor	Transgenesis	HPLC analysis of *MYB10*	Laboratory	(Espley *et al.*,

(*Malus* x d*omestica)*			*domestica* ‘Red field’	regulating anthocyanin accumulation in apple	(Overexpression)	transgenic apple leaves showed a predominant peak of cyanidin-3-galactoside (480 mAU) whereas no peak was observed in control plants		2007)
Apple (*Malus* x d*omestica*	‘M26’	Increased tolerance to salinity stress	*NHX1*	Vacuolar Na^+/H^+ antiporter	Transgenesis (Overexpression)	Na^+ content increased 50% in roots and decreased 27% in leaves, K^+ increased 30% in roots and 50% in leaves when transgenic plants were grown at 150 mM NaCl (relative to WT plants grown under the same conditions)	Laboratory	(Li *et al.*, 2010)
Apple (*Malus* x d*omestica*	‘Gala’ and ‘Golden Delicius’	Reduced susceptibility to *E. amylovora*	*DIPM4*	Susceptibility factor that interacts with a disease-specific gene of *E. amylovora*	Targeted mutagenesis (CRISPR/Cas9)	Necrosis was reduced on average 50% in both apple varieties relative to their controls	Laboratory	(Pompili *et al.*, 2020)
Apple (*Malus* x d*omestica*	‘Gala’	Floral initiation	*TFL1*	Floral repressor	Targeted mutagenesis (CRISPR/Cas9)	93% of the transgenic lines flowered within 3 to 6 months	Laboratory	(Charrier *et al.*, 2019)
Pear (*Pyrus communis*)	‘Conference’	Floral initiation	*TFL1*	Floral repressor	Targeted mutagenesis (CRISPR/Cas9)	Early flowering (within 4-12 months) was observed in 9% of transgenic lines	Laboratory	(Charrier *et al.*, 2019)
Vitis berlandieri x Vitis rupestris	‘Richter’	Increased tolerance to salinity and oxidative stress	*Fer* from *Medicago sativa*	Ferritin is an iron storing protein	Transgenesis (Overexpression)	Photosynthesis loss was 50% in transformed lines vs. 65% in WT. Under oxidative stress, the photosynthesis loss was 45% in transformed lines vs. 79% in WT.	Laboratory	(Zok *et al.*, 2010)
Cucumber (*Cucumis sativus*)	N.A.	Gynoecism	*WIP1*	Inhibitor of carpel development	Targeted mutagenesis (CRISPR/Cas9)	Mutants have 7 times more females flowers than WT	Laboratory	(Hu *et al.*, 2017)
Grapefruit (*Citrus paradisi*)	‘Ruby’, ‘Red’, ‘Rio’ and ‘Duncan’	Increased resistance to Citrus tristeza virus (CTV)	*Ctv-R* from *P. trifoliata*	CTV resistant gene	Transgenesis (Overexpression)	Presence of the virus was not detected by Northern blotting 16 and 24 weeks after inoculation	Laboratory	(Rai, 2006)
Lemon (*Citrus limon*)	‘Femminello siracusano’	Increased resistance to *Botrytis cinerea*	*Chit42* from *Trichoderma harzianum*	Endochitinase which hydrolyses chitin	Transgenesis (Overexpression)	For *B. cinerea*, transgenic plants had reduced disease diffusion (40%-60%) compared to WT (90%)	Laboratory	(Gentile *et al.*, 2007)
Sweet orange (*Citrus sinensis*)	‘Hamlin’	Increased resistance to *Xanthomonas axonopodis pv. citri*	*hrpN* from *E. amylovora*	Harpin protein which elicits the hypersensitive response and systemic acquired resistance in plants	Transgenesis (Overexpression)	Disease severity of lines transgenic lines was reduced up to 79% relative to WT	Laboratory and greenhouse	(Barbosa-Mendes *et al.*, 2009)
Sweet orange (*Citrus sinensis*)	‘Natal’, ‘Pera’, ‘Valencia’	Increased resistance to *Xanthomonas citri subsp. citri*	*attA* from *Trichoplusia Ni*	Antimicrobial peptide that increases membrane permeability in Gram negative bacteria	Transgenesis (Overexpression)	In transgenic plants, the diseased leaf area ranged from 0.16 to 0.30% opposed to 0.72% on control plants	Laboratory	(Cardoso *et al.*, 2010)
Sweet orange (*Citrus sinensis*)	‘Anliucheng’	Increase resistance to *X. axonopodis* pv. *citri*	*SPDS1* from *Malus* x *domestica*	Spermidin synthase involved in polyamines biosynthesis	Transgenesis (Overexpression)	Putrescine content increased 200% in transgenic plants relative to	Laboratory	(Fu *et al.*, 2011)

						WT. Disease index of WT was 55 times higher than in transgenic plants		
Grapefruit (*Citrus paradisi*)	'Duncan'	Increased resistance to *X. citri subsp. Citri*	*LOB1*	Susceptibility gene for citrus canker	Targeted mutagenesis (CRISPR/Cas9)	No canker symptoms were observed on 66% of transgenic plants at 4 days post-inoculation, whereas WT plants showed severe disease symptoms.	Laboratory	(Jia *et al.*, 2017)
Melon (*Cucumis melo*)	'Prince'	Resistance to Cucumber green mottle mosaic virus (CGMMV)	*DR* from Cowpea mosaic virus (CPMV)	Direct repeat (DR) of the movement protein (MP) of CPMV	Transgenesis (Gene silencing)	Transgenic lines had 25% less CGMMV and did not show symptoms	Laboratory	(Emran *et al.*, 2012)
Grapevine (*Vitis vinifera*)	'Portan'	Response to hypoxia	*Adh2* from *Vitis vinifera*	Alcohol dehydrogenase 2 involved in plant development and response to abiotic stress	Transgenesis (overexpression and gene silencing)	Under hypoxia, ADH activity in sense lines was 5-times higher than in control and antisense lines. Under aerobic conditions, terpene emission increased 5-foldin sense lines than in control and antisense lines	Laboratory	(Tesniere *et al.*, 2006)
Grapevine (*Vitis vinifera*)	'Maccabeu', 'Chardonnay', 'Syrah', 'Portran', and 'Tempranillo'	Increased anthocyanin content	*VlmybA 1-2* from *Vitis labruscana*	Transcription factor involved in anthocyanin biosynthesis	Transgenesis (overexpression)	Transgenic lines had 35% more malvidin in roots than WT	Laboratory	(Cutanda-Perez *et al.*, 2009)
Grapevine (*Vitis vinifera*)	'Centennial Seedless'	Increased tolerance to cold stress	*DREB1b* from *A. thaliana*	Cold-inducible transcription factor for COR genes	Transgenesis (overexpression)	After cold treatment 26% of transgenic plants wilted, compared with 98% in WT. 95% recovered, compared with 2% in WT	Laboratory	(Jin *et al.*, 2009)
Kiwifruit (*Actinidia deliciosa*)	N.A.	Resistance to fruit-piercing moth (*Oraesia excavate*)	*SbtCry1Ac* (Synthetic chimeric)	CrylAc insecticidal protein	Transgenesis (overexpression)	Control plants had 63.4% leaf damage, whereas transgenic plants had 21.3% leaf damage	Laboratory	(Zhang, Liu and Liu, 2015)
Pear (*Pyrus communis*)	'Ballad'	Tolerance to salinity, osmotic and copper stress	*SPDS1* from *Malus ×domestica*	Spermidine synthase that catalyses the conversion of putrescine to spermidine *SPDS1* is also involved abiotic stress response	Transgenesis (overexpression)	Weight of transgenic shoots was similar under salinity, and copper stress and control conditions, whereas in control shoots it decreased 60%. Osmotic stress reduced weight 30% in transgenic shoots and 100% in control shoots.	Laboratory	(Wen *et al.*, 2008)
Pear (*Pyrus communis*)	'La France' and 'Ballade'	Floral initiation	*FT* from *Citrus unshiu*	*FLOWERING LOCUS T* is a floral pathway integrator	Transgenesis (overexpression)	Floral buds developed in 50% of transgenic plants 4-weeks after transferred to micropropagation medium, whereas control plants did not develop any floral bud	Laboratory	(Matsuda *et al.*, 2009)
Plum (*Prunus domestica*)	'Stanley'	Increased resistance to Plum pox virus (PPV)	*h-UTR/P1*	PPV-derived hairpin construct which confers PPV resistance	Transgenesis (gene silencing)	Out of a total of 96 successful grafts, 6 apexes did not contain PPV at the end of the experiment (6.2%)	Laboratory	(Monticelli *et al.*, 2012)
Plum (*Prunus*	'Angeleno' and	Increased resistance	Hairpins *eIFiso4E,*	Eukaryotic translation	Transgenesis	A transgenic line tested	Laboratory	(Rubio *et al.*,

salicina)	'Larry-Ann'	to Plum pox virus (PPV)	*eIFiso4G, eIFiso4G11,* and *eIFiso4G10*	initiation complex indispensable for viral infection.	(gene silencing)	negative for PPV infection after PPV challenge for five vegetative cycles		2019)
Tomato (*Solanum lycopersicum*)	'Money Maker'	Resistance to a range of phytopathogenic bacteria	*EFR* from *Arabidopsis thaliana*	Pattern-recognition receptor that detects microbes by recognizing conserved pathogen-associated molecular patterns	Transgenesis (overexpression)	Bacterial population was lower in *EFR*- mutants (6.5 log CFU/cm^2) than in WT plants (8 log CFU/ cm^2)	Laboratory	(Lacombe *et al.*, 2010)
Tomato (*Solanum lycopersicum*)	'M82'	Fruit size and inflorescence	Cis-regulatory element of *CLV3*	CLAVATA controls locule number and fruit size	CRISPR/Cas9	Flowers of CLV3-mutants had more sepals, petals, stamen, and fruit locule number than WT (100%, 60%, 140% and 400% increase, respectively)	Laboratory	(Rodríguez-Leal *et al.*, 2017)
Tomato (*Solanum lycopersicum*)	N.A.	Increased tolerance to drought	***NPR1***	Non-expressor of pathogenesis-related gene is involved in plant defense against pathogens	CRISPR/Cas9	Survival rate of *NPR1* mutants was 20 to 50% after drought, whereas 80% of WT survived. Stomatal aperture was 3.5 times higher in mutants than in WT	Laboratory	(Li *et al.*, 2019)
Walnut (*Juglans hindsii* x *Juglans regia*)	'J1' and 'RR4'	Increased crown gall and nematode resistance	*A. tumefaciens* self-complementary *iaaM* and *ipt. Pratylenchus vulnus* self-complementary *Pv010. A. rhizogenes rolABC*	Resistance to *A. tumefaciens*, *P. vulnus*, and *A. rhizogenes*	Transgenesis (gene silencing)	Transgenic plants showed 79% less nematodes per root after *in vitro* co-culture than non-transformed plants and complete crown gall control	Laboratory	(Walawage *et al.*, 2013)
Apple (*Malus* x *domestica)*	'Greensleeves'	Reduced ethylene production to modify fruit flavor	*ACS* *ACO*	1- aminocyclopropane-1-carboxylic acid synthase and oxidase, enzymes responsible for ethylene biosynthesis	Transgenesis (Gene silencing)	Ethylene production decreased 60% relative to WT. Firmness increased 20% and color hue angle increased 4%. Soluble solids and acidity were comparable to those in WT	Laboratory and Field trials	(Dandekar *et al.*, 2004)
Tomato (*Solanum lycopersicum*)	'Micro-Tom' and 'Ailsa Craig'	Parthenocarpy	*IAA9*	Negative regulator of parthenocarpy	CRISPR/Cas9	WT fruits had 15 seeds whereas transgenic lines had 0-4 seeds per fruit.	N.A.	(Ueta *et al.*, 2017)
Banana (*Musa spp.*)	'Grand Naine' and 'Rasthali'	Increased β-carotene content	*LCYε*	Lycopene ε-cyclase, which converts lycopene into α-carotene	Targeted mutagenesis (CRISPR/Cas9)	Edited lines accumulated β-carotene up to 6-fold	Net-house	(Kaur *et al.*, 2020)
Cucumber (*Cucumis sativus*)	'Ilan'	Increased resistance to Cucumber vein yellowing virus (CVYV), Zucchini yellow mosaic virus (ZYMV), Papaya ringspot virus (PRSV) and Cucumber mosaic virus (CMV)	*elF4E*	Eukaryotic translation initiation factor 4E, which interacts with viral encoded proteins and promotes virus infectivity	Targeted mutagenesis (CRISPR/Cas9)	No symptoms of CVYV, ZMYV, and PRSV infection were developed in transgenic plants (0/20) whereas 60 of 60 WT plants showed symptoms. High susceptibility to CMV infection was observed in both transgenic (11/11) and WT (9/9)	Net-house	(Chandrasekaran *et al.*, 2016)

[a] Direct DNA delivery

N.A. Not available
